# Genomic Approaches Uncover Increasing Complexities in the Regulatory Landscape at the Human SCL (TAL1) Locus

**DOI:** 10.1371/journal.pone.0009059

**Published:** 2010-02-05

**Authors:** Pawandeep Dhami, Alexander W. Bruce, Johanna H. Jim, Shane C. Dillon, Amanda Hall, Jonathan L. Cooper, Nicolas Bonhoure, Kelly Chiang, Peter D. Ellis, Cordelia Langford, Robert M. Andrews, David Vetrie

**Affiliations:** 1 The Wellcome Trust Sanger Institute, Hinxton, United Kingdom; 2 Section of Pathology and Gene Regulation, Division of Cancer Sciences and Molecular Pathology, University of Glasgow, Glasgow, United Kingdom; Institute of Genetics and Molecular and Cellular Biology, France

## Abstract

The SCL (TAL1) transcription factor is a critical regulator of haematopoiesis and its expression is tightly controlled by multiple *cis*-acting regulatory elements. To elaborate further the DNA elements which control its regulation, we used genomic tiling microarrays covering 256 kb of the human SCL locus to perform a concerted analysis of chromatin structure and binding of regulatory proteins in human haematopoietic cell lines. This approach allowed us to characterise further or redefine known human SCL regulatory elements and led to the identification of six novel elements with putative regulatory function both up and downstream of the SCL gene. They bind a number of haematopoietic transcription factors (GATA1, E2A LMO2, SCL, LDB1), CTCF or components of the transcriptional machinery and are associated with relevant histone modifications, accessible chromatin and low nucleosomal density. Functional characterisation shows that these novel elements are able to enhance or repress SCL promoter activity, have endogenous promoter function or enhancer-blocking insulator function. Our analysis opens up several areas for further investigation and adds new layers of complexity to our understanding of the regulation of SCL expression.

## Introduction

Understanding the molecular events which occur as stem cells differentiate into committed cell lineages is a fundamental issue in cell biology. It has been shown that the SCL (TAL1) gene is central to the mechanisms whereby pluripotent stem cells differentiate into haematopoietic stem cells (HSCs) that give rise to the various blood lineages. While this process is thought to be tightly regulated at the level of gene expression, the exact ways in which SCL helps direct this process are not well understood.

The SCL gene encodes a bHLH transcription factor (TF) which is normally expressed in blood, endothelium and various areas of the central nervous system. In the haematopoietic compartment, SCL is expressed in multipotent haemangioblasts and HSCs, in erythroid and megakaryocytic progenitors, and mast cells. SCL is required during embryonic stem (ES) cell differentiation [1], [2] and for the establishment of all haematopoietic lineages [3]–[6], but is not essential for self-renewing of HSCs [7]. Recently it has been shown that expression of either SCL or the highly related bHLH TF Lyl1 is required for maintenance of HSCs [8]. SCL expression is required for normal differentiation of erythroid and megakaryocytic lineages [7], [9], whereas inappropriate expression during T-cell differentiation leads to T-cell acute lymphoblastic leukemia (T-ALL) [10].

Evidence suggests that the transcriptional regulation of SCL is tightly controlled in all of its biological roles. SCL is transcribed from two promoters (1a and 1b) located at the 5′ end of the gene [11], and an additional promoter within exon 4 [12]. The analysis of accessible chromatin using DNase I hypersensitive assays, comparative sequence analysis and chromatin immunoprecipitation (ChIP) in combination with real-time PCR has led to the identification of additional regulatory elements [13]–[15] which have activity in transfection assays or direct SCL expression into specific compartments of its normal expression pattern in transgenic mice. However, the elaborate nature of the *cis*- and *trans*-acting events which control the concerted activity of all of these elements remains unclear.

The use of genomic microarrays in combination with methods such as chromatin immunoprecipitation (ChIP-chip) [16]–[18], are powerful high-throughput approaches which allow events associated with the transcription of genes and the activity of their regulatory sequences to be elucidated *in vivo*. In the present study, a tiling path genomic microarray spanning 256 kb of the human SCL locus was constructed and used in combination with ChIP and chromatin accessibility assays to study the full complement of *cis*- regulatory elements associated with the transcriptional regulation of SCL and its flanking genes in haematopoietic cells. From this analysis, known SCL regulatory regions and a number of novel regulatory elements, which lay both upstream and downstream of SCL, were identified and further characterized. The identification and characterisation of these regulatory elements, and their relevance to SCL expression and regulatory control are discussed.

## Results

We constructed a tiling microarray across 256,636 bp of the genomic region containing the human SCL locus at an average resolution of 458 bp. The tiling array encompassed SCL and all of its known regulatory sequences, and the neighbouring genes MAP17 (PDZK1IP1) and SIL (STIL), and the 5′ end of the housekeeping KCY (CMPK, UCK) gene, all of which are expressed during haematopoiesis [14]. We also included genomic regions covering the genes for CYP4A22 and most of CYP4AZ1 located downstream of MAP17, which are not appreciably expressed during haematopoiesis [14]. The hybridization of fluorescently-labelled unamplified ChIP DNAs, coupled with a highly sensitive and quantitative array platform [19], allowed us to identify reproducibly (Figure S1), and validate with real-time PCR (Figure S2), a wide range of regulatory features across the SCL locus as described below.

### Histone Modifications Define Known and Novel Regions of Regulatory Function

**Figure 1:**
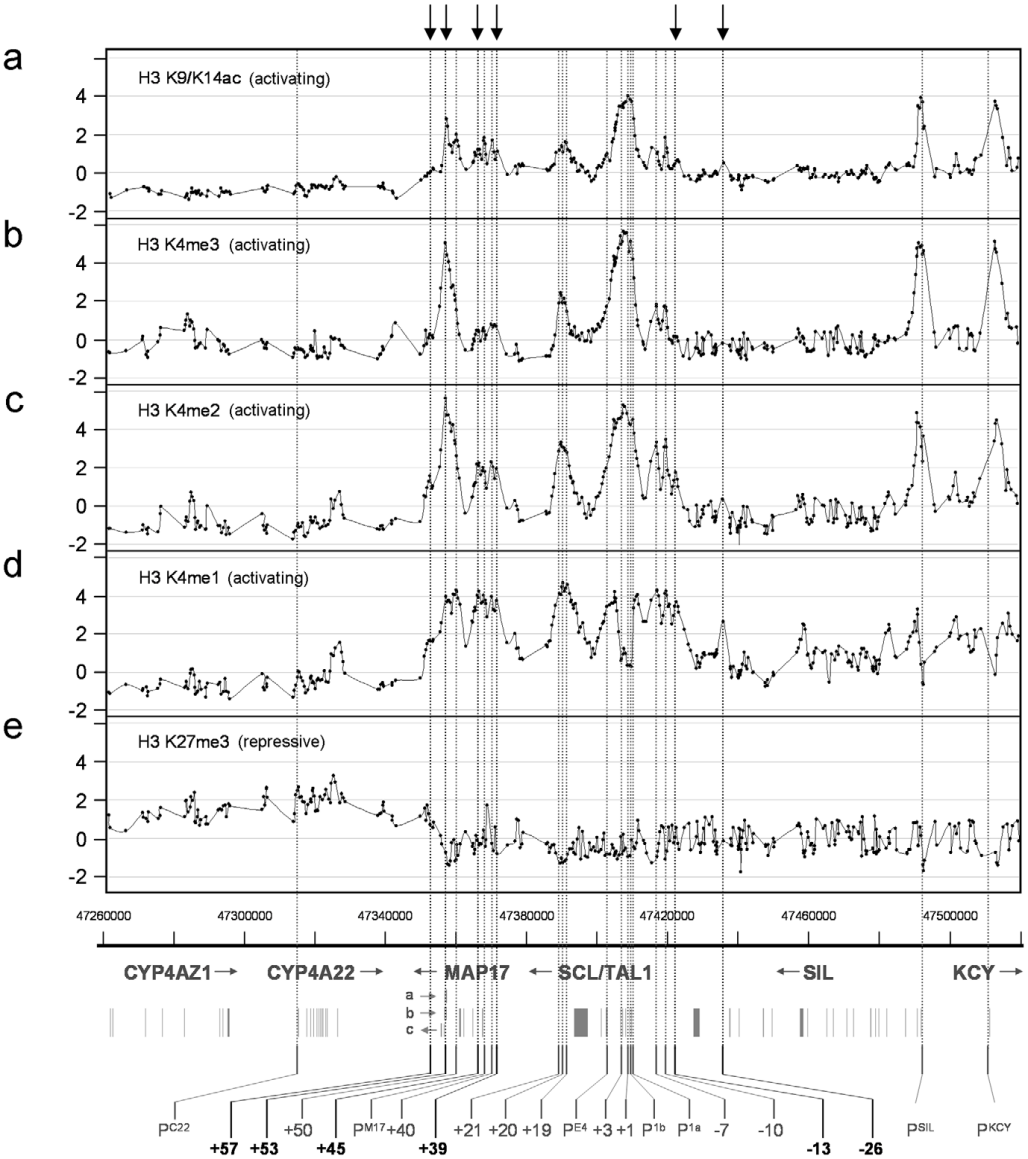
Histone H3 modification profiles across the human SCL locus in the K562 cell line. The modifications studied are named at the left of each panel. Dots on the solid joined-up lines represent the data obtained for each genomic tiling array element. In each panel, the x-axis is the genomic sequence co-ordinate (NCBI build 35) and the y-axis is the enrichment obtained in ChIP-chip assays expressed in log_2_ scale. Schematic diagram at the bottom of the figure shows the genomic organisation of SCL and its neighbouring genes. Exons are shown as vertical blocks with gene names and direction of transcription shown above. Transcripts denoted by a,b and c refer to transcripts of unknown function (see also text). Vertical lines at the bottom (with dotted lines through all the panels) show the location of known and novel regulatory regions at the SCL locus. Promoters are denoted by P. Other nomenclature refers to the distance in kb from SCL pro1a (P^1a^). Novel regions are highlighted in bold at the bottom and with arrows at the top of the figure.

It is well established that histone H3 post-translational modifications play an intrinsic role in transcriptional regulation and mark both active and silent regulatory sequences and/or entire gene loci [20]–[26]. To delineate novel regulatory elements across the SCL locus, we profiled activating and repressive histone marks in the SCL-expressing K562 cell line (Figure 1). K562 cells represent a well-characterized model of the erythroid lineage and show a high concordance with respect to ChIP-chip data from primary mammalian erythroid cells at both the gene level and genomewide [24], [27]-[31].

We identified a number of regions of clearly discernable enrichments for the activating modifications histone H3 K9/K14 di-acetylation (H3 K9/K14ac) and histone H3 K4 methylation (H3 K4me1/me2/me3), many of which marked regulatory regions which had been studied previously at the human SCL locus. These included the promoters of the KCY, SIL and SCL genes that are expressed in K562 (as determined by Affymetrix GeneChip expression profiling [24]), and at sites within the SCL transcript known to have promoter or enhancer activity [the exon 4 promoter (P^E4^) and the +1, +3 regions]. Enrichments were also found at the SCL haematopoetic stem cell enhancer (+19/+20/+21) [14], [32], the SCL erythroid enhancer (+50) [14], [15], the MAP17 promoter and its enhancer (P^M17^ and +40 respectively) [31], and the −7 and −10 regions [14], [33], [34]. All these regions are named according to their distances downstream (+) or upstream (−) in kb from the human SCL pro1a (P^1a^ in Figure 1).

In addition, we detected a number of peaks of H3 K4 methylation and H3 K9/K14 diacetylation across the SCL locus which had not been identified by any means in previous studies. The most prominent of these was located 53 kb downstream of SCL P^1a^ (+53) and showed particularly high levels of H3 K4me3. This region is also associated with a number of novel transcripts of unknown function (denoted a, b and c in Figure 1). We also found sites of enrichments, most prominent with H3 K4me2 and H3 K4me1, at −13, and −26, upstream of SCL P^1a^, and at +39, +45, and +57. The latter one of these, +57, was also associated within a region which showed a transition from low levels of the repressive mark H3 K27me3 over the transcriptionally-active SCL, MAP17, SIL and KCY genes to higher levels over the silent CYP4AZ1 and CYP4A22 genes (Figure 1). Transitions in levels of H3 K27me3 have previously been associated with CTCF-binding and insulator activity, demarcating boundaries between transcriptionally-active and silent gene loci [35]. These patterns of activating and repressive histone marks are consistent with expression patterns of genes at the SCL locus [14]. More importantly, they define discrete blocks of active chromatin and repressive chromatin, demarcated at, or near, +57 − suggesting that this region may define a putative insulator.

### The Transcriptional Machinery, CTCF and TFs Define Regions with Regulatory Function

We defined further both known and novel regulatory sequences at the SCL locus by associating them with a number of biologically important proteins involved in transcriptional regulation. These included RNA polymerase II (PolII) and TAFII 250 (also know as TAF1), the insulator protein CTCF [36], [37], and transcription factors which have important roles in haematopoeisis.

**Figure 2:**
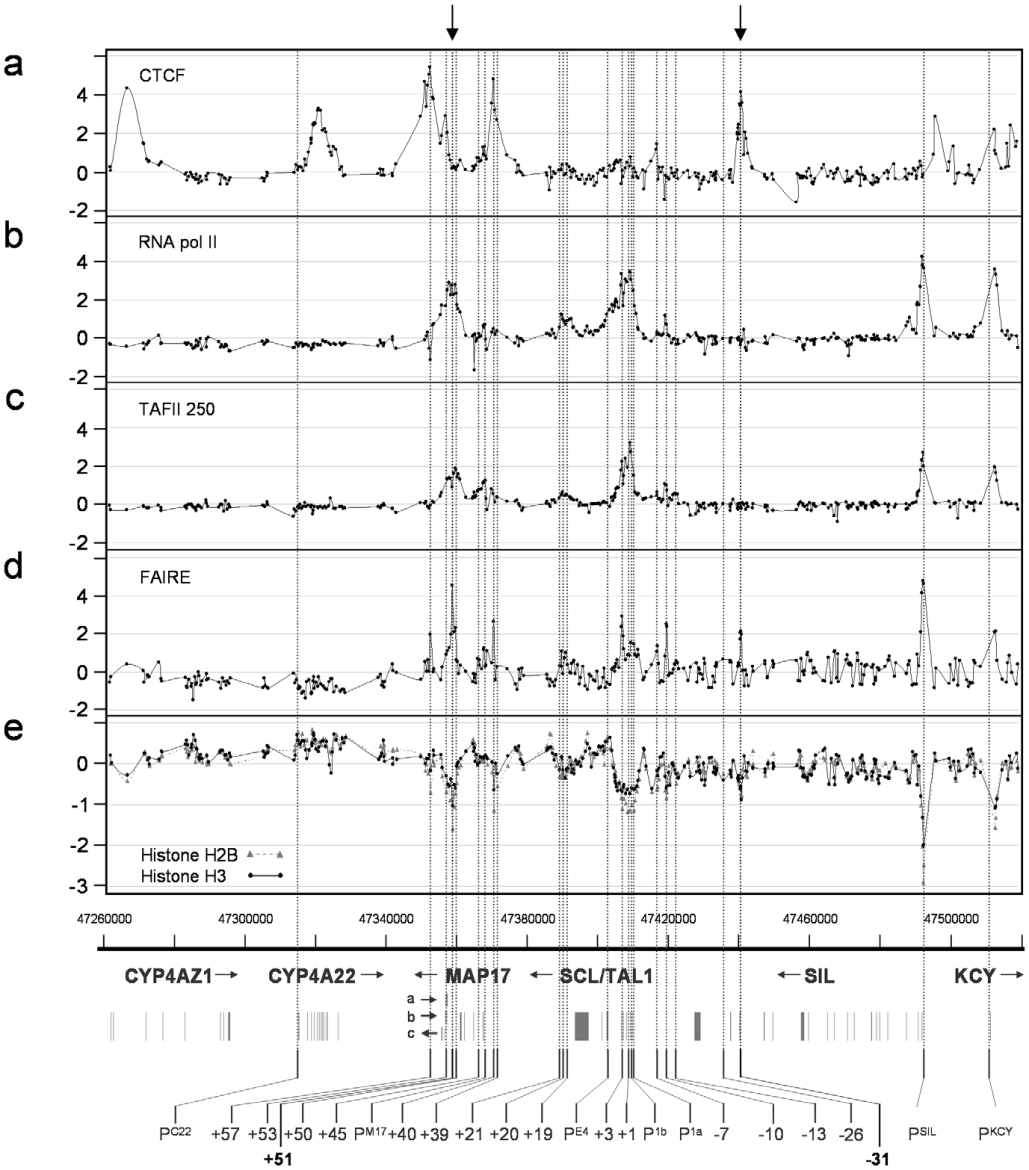
CTCF, PolII and TAFII 250 binding, chromatin accessibility and nucleosome density profiles across the human SCL locus in the K562 cell line. Assays used are named at the left of each panel. Dots on the solid joined-up lines represent the data obtained for each genomic tiling array element. In panel E, the profiles for histone H2B and histone H3 are shown as dotted joined-up line with triangles and solid joined-up lines with dots for H2B and H3 respectively. All other aspects of the figure are as in Figure 1.

As we anticipated, prominent peaks of enrichment for PolII and TAFII 250 were found at or near the promoters of SCL, SIL and KCY (Figure 2) as these genes are transcribed in K562 to relatively high levels (data not shown). Less significant peaks were associated with the MAP17 promoter (P^M17^), the transcript from which is expressed at significantly lower levels [14]. Both PolII and TAFII 250 enrichments extended from SCL P^1a^ to the +3 region, with PolII extending to SCL P^E4^. We also detected peaks of PolII and TAFII 250 binding at other known and putative SCL regulatory elements: at the stem cell enhancer, the −10 region, and across 3 kb spanning the erythroid enhancer (+50 to +53). The binding of the PolII machinery at enhancers is well-known [38].

By contrast, CTCF showed patterns of binding consistent with H3 K27me3 patterns (see above) and/or its known role as an insulator which defines transcriptional domains [35], [36]. Prominent peaks of enrichment were found at a novel region between SCL and SIL 31 kb upstream of SCL pro1a (−31) and at +57, between the erythroid enhancer at +51 and CYP4A22. Similarly, prominent peaks were found within CYP4A22, CYP4AZ1 and between or within SIL and KCY. CTCF was also found bound at +53, the MAP17 enhancer (+40), and the −7 region. Three of these sites (+57, +53 and +40) were also confirmed experimentally or predicted in other studies based on the consensus binding motif of CTCF [36].

The transcription factors GATA1, LMO2, E2A, LDB1 and SCL itself are known to associate in a DNA-binding complex in erythroid cells [39], [40] which regulates gene expression [41]. Regions of statistically significant enrichments (Table S1) for all or some of these TFs were found at a number of SCL regulatory sequences including the SCL P^1a^, P^E4^, −7 and −10 regions and most prominently all were found bound at +51 (Figure S3), one kb downstream from the known peaks of histone H3 K9/K14ac [14] and H3 K4me1/me2 or me3 associated with the erythroid enhancer at +50.

### Chromatin Architecture Facilitates Accessibility to Regulatory Regions

It has been demonstrated that the detection of decreased levels of nucleosomes (i.e., nucleosome depletion) [24], [25], [42]–[45] or an inability to detect sub-types of nucleosomes (using standard ChIP methods) [46] may be a general feature of active genes and their active regulatory sequences in eukaryotic genomes. We found that nucleosome architecture at the human SCL locus was in agreement with these models. ChIP-chip profiles in K562 cells for the nucleosomal components histone H3 and histone H2B were compared with the results of our FAIRE chromatin fractionation assays (Figure 2). FAIRE assays allow DNA segments which are less readily cross-linked with proteins after formaldehyde treatment (i.e., regions of accessibility or DNase I hypersensitivity) to be physically separated from bulk cross-linked chromatin using phenol-chloroform fractionation [47], [48]. FAIRE peaks across the SCL locus coincided with DNase I hypersensitivity sites found in K562 [31], [49], and there was a very high inverse correlation between nucleosome density and FAIRE (R = −0.861) across the SCL locus (Figure S4), demonstrating that FAIRE peaks identified regions of accessible chromatin which had lower levels of nucleosomes.

We also found that both known SCL regulatory elements and novel regions identified in the present study exhibited lower levels of nucleosomes and higher levels of chromatin accessibility in K562 cells. These included the promoter regions of the KCY, SIL and SCL genes and within transcribed sequences at the 5′ end of SCL. Low levels of nucleosomes were also found at the erythroid enhancer (at +51 but low nucleosome levels extended from +50 to +53), the +57 region, the MAP17 enhancer, and the −7, −10 and −31 regions. These novel regions were also bound by components of the general transcriptional machinery and/or TFs, thus possessing similar structural and functional features as known SCL regulatory regions. Although these regions showed lower levels of nucleosome occupancy, they also showed high levels of activating histone modifications (H3 K4me1,2 or 3 and H3 K9/K14ac) – suggesting that whilst there were fewer nucleosomes in these regions, the nucleosomes which were localised there had very high propensities for being modified.

### Structural and Functional Analysis of Novel Regulatory Elements

We used a number of computational and experimental approaches to further assess the regulatory nature of the novel regions we had identified. This analysis included the novel regions that were either upstream or downstream of SCL and up to and including the +57 and −31 regions respectively. Both +57 and −31 bind CTCF and define an 88 kb regulatory domain containing SCL, MAP17 (thought to be co-regulated with SCL [14]) and all known SCL regulatory elements. None of the nearby genes outside of this domain are thought to share regulatory elements with SCL, suggesting that CTCF at these sites marks the location of insulators [50]. We examined seven regions in this interval which had previously not been analysed in human haematopoietic cell types. These included +57, +53 and +51 located near the +50 erythroid enhancer, and −7, −10, −13 and −31 upstream of SCL.

**Figure 3:**
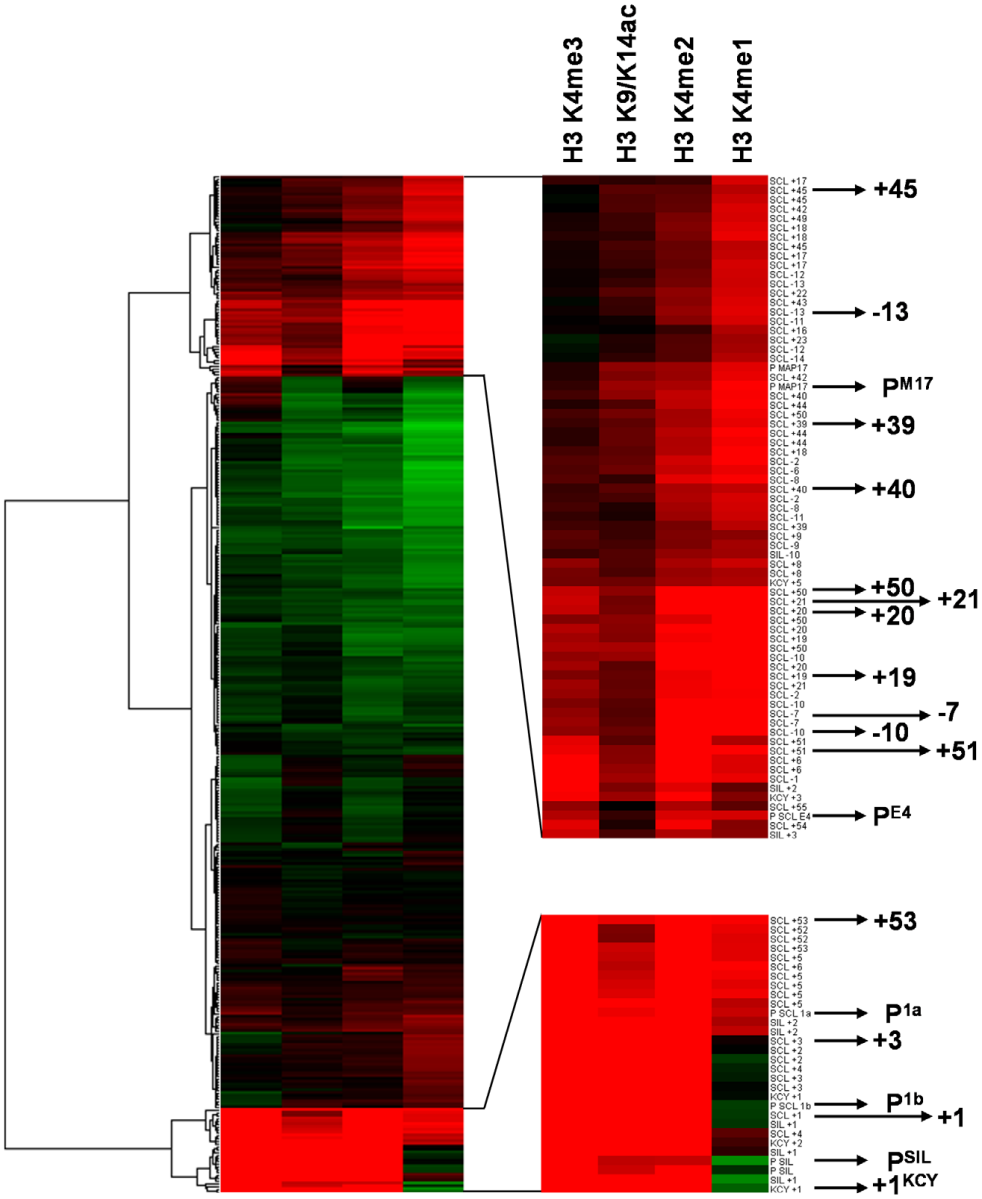
Treeview diagram of hierarchical clustering of genomic tiles across the human SCL locus for histone modifications in the K562 cell line. Each tile is represented by a horizontal bar and is shaded in red scale or green scale to denote level of enrichment or depletion respectively. The tree diagram is shown to the left of the figure. Two branches of the tree and the histone modifications studied are shown in the enlarged panel to the right of the figure. Each genomic tile in the enlarged panel is named according to its distance in kb relative to SCL pro1a or to the promoter of its closest known gene. Tiles representing SCL known and novel regulatory elements are denoted by an arrow and highlighted in bold.

It has been established by us and others that active promoters and enhancers can be discriminated from each other based on levels of activating histone H3 modifications (H3 K9/K14ac and H3 K4me1,2,3) [22], [24]–[26], [51]. With this in mind, we used hierarchical clustering of our data across the SCL locus for these modifications as a means of attributing putative promoter and enhancer function to these novel regions (Figure 3). Microarray tiles containing DNA sequences for known regulatory elements and novel ones formed two distinct clusters with differing histone modification signatures. The first cluster showed hallmarks of promoters [24], [25] with high levels of H3 K9/K14ac and H3 K4me2,3 and lower levels of H3 K4me1. Contained within this cluster were microarray tiles for promoters and adjacent sequences for SCL, KCY and SIL, the +3 region, whose proposed function is consistent with promoter activity [52], and the +53 region located in the vicinity of novel transcripts.

The second cluster showed signatures of active enhancers [24], [25] with high levels of H3 K4me1,2 and lower levels of H3 K9/K14ac and H3 K4me3. Contained within this cluster were microarray tiles for known SCL and MAP17 enhancers (+19/+20/+21, +40 and +50), a number of novel regions (−7, −10, −13, +39, +45, +51) and two promoters which were either inactive or had low activity in K562 (SCL P^E4^ and P^M17^). Thus, these novel regions had attributes of active enhancers in K562 and low activity promoters also had an active enhancer-like histone modification pattern. However, neither the −31 or +57 regions, which both bind CTCF, were found in this cluster suggesting that these regions may be functionally distinct from active enhancers.

**Figure 4:**
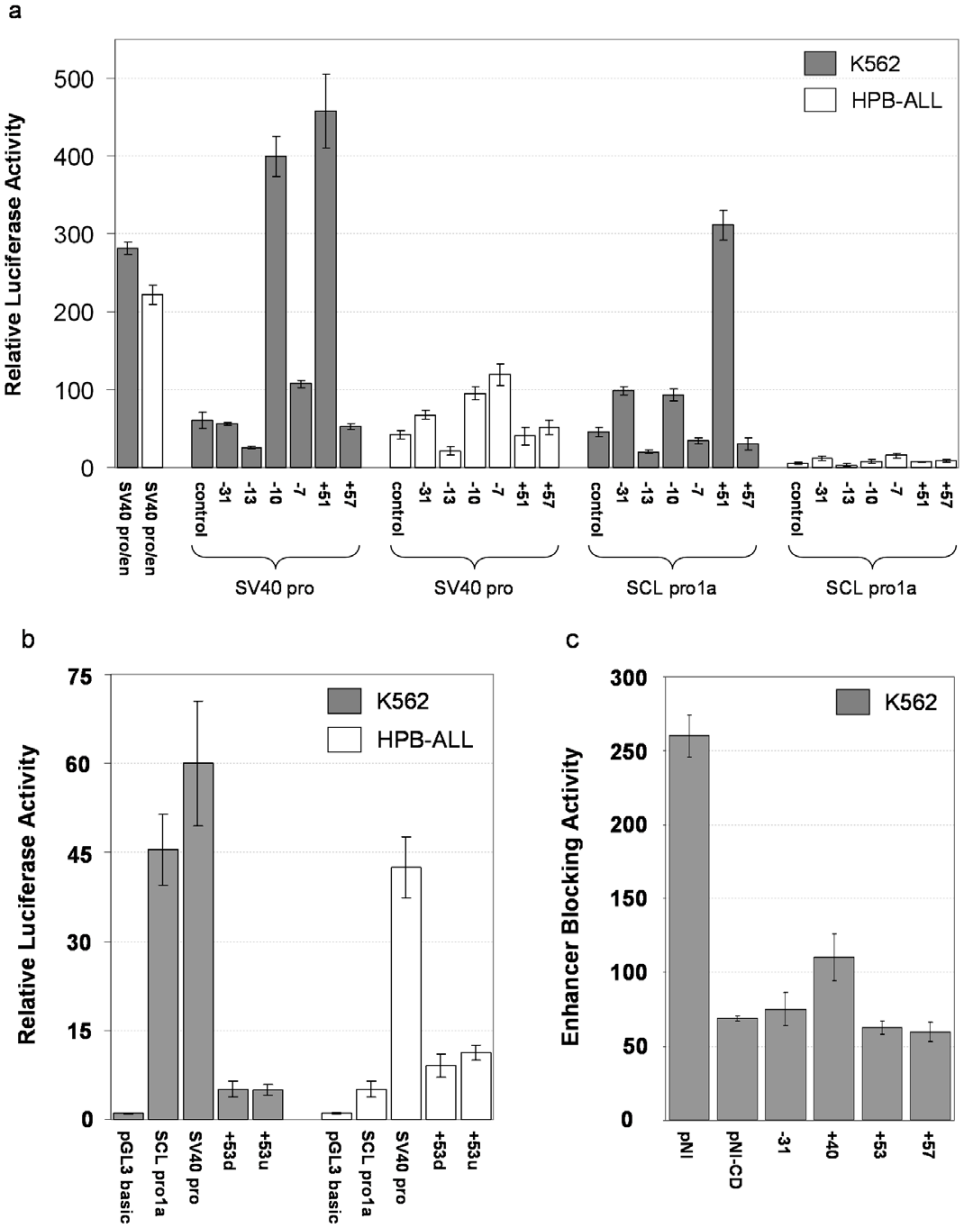
Transient reporter assays of novel regulatory elements at the human SCL locus. **A.** Enhancer/repressor activity of six novel regulatory elements in K562 (erythroid) and HPB-ALL (lymphoid) cell lines. The y-axis shows the fold increase/decrease in luciferase activity relative to the pGL3 basic negative control construct (not shown). The x axis shows constructs under the control of the SV40 promoter (SV40 pro) or the promoter 1a of SCL (SCL pro1a). All constructs under the control of these two promoters are named according to the region being tested based on their distance in kb from the SCL pro1a (−  =  upstream; +  =  downstream). The SV40pro and SCLpro1a control lanes shown were derived from transfections with constructs containing a sequence having no regulatory function cloned into the *Bam*HI site. Luciferase expression for the SV40 promoter in combination with the SV40 enhancer (SV40 pro/en) is also shown as a positive control. Standard error bars are shown. **B.** Promoter assays of the +53 region in K562 (erythroid) and HPB-ALL (lymphoid) cell lines. The y-axis shows the fold increase/decrease in luciferase activity relative to the pGL3 basic negative control construct. The x axis shows luciferase expression driven by pGL3 basic, SCL pro1a only (SCL pro1a positive control), SV40 pro (SV40 promoter positive control), and the two +53 constructs (SCL+53u and SCL+53d). The +53 region was cloned in one (u) or the other (d) orientation. Standard error bars are shown. **C.** Enhancer blocking assays of regions at the SCL locus which bind CTCF in the K562 cell line. The y-axis shows enhancer blocking activity (G418-resistant colony counts) for the pNI and pNI-CD (negative and positive controls) and four putative insulator regions from the human SCL locus. Insulator constructs are named according to the region being tested based on their distance in kb from the SCL pro1a (−  =  upstream; +  =  downstream). Standard error bars are shown. Note: Enhancer blocking assays were performed only in the K562 cell line as expression of the pNI-based vector is regulated by the murine HS2 β-globin enhancer which is active only in erythroid cells.

Given that several of the novel regions had epigenetic hallmarks of either promoter and enhancer activity, we analysed a panel of transient luciferase reporter constructs in K562 (erthyroid/SCL +) and HPB-ALL (lymphoid/SCL -) to ask whether these novel sequences could enhance or drive gene expression *in vivo* and whether their effects were lineage-specific or specific to SCL regulation. We tested whether the +53 region had promoter activity, and whether the six other regions (+57, +51, −7, −10, −13, and −31) could enhance luciferase expression under the control of the SCL pro1a and SV40 promoters (Figure 4). Of those regions assayed for enhancer activity, four were able to increase luciferase expression levels under the control of the SCL pro1a and/or SV40 promoters in either K562 or HPB-ALL (Figure 4a). The +51 and −10 regions showed enhancer activity on both the SV40 and SCL pro1a promoters, whereas −7 and −31 showed enhancer activity on only the SV40 promoter (−7) or only on SCL pro1a respectively (−31). −31, −10 and −7 exhibited enhancer activity in both K562 and HPB-ALL, while +51 was only active in K562, suggesting it had erythroid-specific enhancer activity. The −13 region showed repressor activity on both the SCL pro1a and SV40 promoters by reducing luciferase activity in the range of 42–57% (stastically significant decreases in both K562 and HPB-ALL) indicative of repressor activity in both cell types. However, the +57 region was not able to modulate luciferase expression on either promoter.

The +53 region exhibited low levels of promoter activity in both cell lines and in both orientations when compared to the activity of SCL pro1a and the SV40 promoter at driving luciferase expression (Figure 4b). However, this activity was statistically significant above background, indicative of +53 being a bidirectional promoter. This is consistent with this region co-localising with lowly expressed novel transcripts (see below), and having a histone modification signature of promoters.

We demonstrated that CTCF was bound to −31, +53, +57 (see above and Figure 2). Given the known role of CTCF binding at regions exhibiting enhancer-blocking insulator function [37], we assayed for any enhancing-blocking activity for these three regions as well as a region upstream of the MAP17 promoter which also showed CTCF binding in our analyses, as well as enhancer activity in previous studies [31]. Enhancer-blocking activity was based on the ability of a test sequence being able to block enhancer activity when placed between a known enhancer capable of driving expression of geneticin (G418)-resistance in the K562 erythroid cell line. By measuring the number of K562 colonies obtained for each of the constructs under selection with geneticin, we were able to demonstrate enhancer-blocking activity for all four of the test regions (Figure 4c), consistent with these regions having insulator activity.

Computational analysis of the novel elements we had identified revealed DNA sequence-based features indicative of conserved regulatory function across mammalian species. Novel regions showed computational five-way regulatory potential [53], [54] (data not shown) and some, but not all, showed conservation of relevant DNA sequence motifs. In particular, we identified a number of highly conserved binding motifs in the −13 repressor region, including an 11 bp sequence for the ets family ETV6/7 proteins (Figure S5). ETV6 (TEL1) and ETV7 (TEL2) are transcription factors, expressed during haematopoiesis and have been shown to exhibit strong repressor activity *in vivo* [55], [56]. The +51 region showed a striking conservation of three GATA and other TF binding sites (Figure S6). One of these GATA sites was contained within a 20 bp sequence containing a GATA/E-box composite site which showed the canonical hallmarks of the SCL-containing erythroid complex [39], [40].

Previous work had suggested that the erythroid enhancer, which directs SCL expression during primitive and definitive erythropoiesis [14], [15] was defined at +50 [14]. However, our analysis demonstrates that the 1 kb region adjacent to +50, at +51, contains conserved DNA sequence motifs which are likely to be the core site of binding for the SCL-containing erythroid complex (henceforth, the core erythroid enhancer is renamed the +51 region). This is consistent with recent studies of the murine equivalent of the SCL erythroid enhancer [15]. However, the presence of a bi-directional promoter at +53, highlights the complexity of the regulatory landscape around the erythroid enhancer.

### The Regulatory Complexity at the SCL Erythroid Enhancer

With this is mind, we sought to more accurately define the regulatory environment of the +51 SCL erythroid enhancer in cell lines where it was either active or inactive. Using ChIP-chip, we examined regulatory features across a 4 kb region (+50 to +53) in three non-erythroid haematopoietic cell lines (U937, HL-60 and HPB-ALL) which do not express SCL and compared these features with those found in K562.

**Figure 5:**
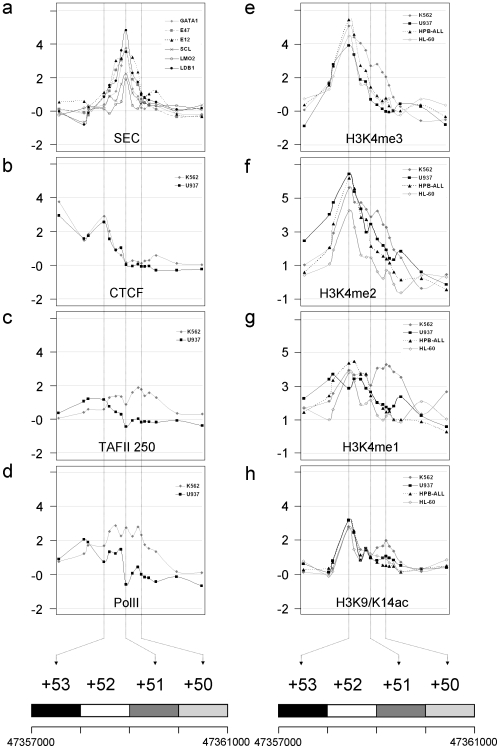
ChIP-chip analysis of the human SCL erythroid enhancer region in SCL-expressing and non-expressing haematopoietic cell lines. **A.** Binding of the SCL-containing erythroid complex (SEC) of transcription factors in the K562 cell line. E47 and E12 are two isoforms of E2A. **B.** CTCF binding in the K562 and U937 cell lines. **C.** TAFII 250 binding in the K562 and U937 cell lines. **D.** PolII binding in K562 and U937 cell lines. **E.** Histone H3 K4me3 in the K562, U937, HPB-ALL and HL-60 cell lines. **F.** Histone H3 K4me2 in the K562, U937, HPB-ALL and HL-60 cell lines. **G.** Histone H3 K4me1 in the K562, U937, HPB-ALL and HL-60 cell lines. **H.** Histone H3 K9/K14ac in the K562, U937, HPB-ALL and HL-60 cell lines. All enrichments obtained in ChIP-chip assays are expressed in log_2_ scale in the figure. Schematic at the bottom shows the 4 kb of genomic DNA surrounding the SCL erythroid enhancer. Genomic sequence co-ordinates are from NCBI build 35. Numbering of the blocks of DNA (with dotted lines through all the panels) refers to the distance in kb from SCL pro1a. The vertical dotted lines are positioned at the mid-point of each of the 1kb blocks and do not define positions of regulatory regions *per se*.

The SCL-containing erythroid complex (SEC) was bound at +51 in K562 (Figure 5a) and not in the other cell lines (U937, HL60, HPB-ALL) (not shown). We also confirmed that the SEC was present at +51 in a second erythroid cell type HEL 92.1.7 which expressed SCL [14] (Figure S7). CTCF was bound to the +53 region in all these cell lines (K562 and U937 shown in Figure 5b), approximately 500 bp from the region shown to have bi-directional promoter activity. TAFII 250 and PolII showed binding patterns consistent with the location of the bi-directional promoter at +53 in all of the cell lines (K562 and U937 shown in Figure 5c and 5d respectively). In K562, PolII was also bound to the +51 erythroid enhancer, along with TAFII 250 at +50. These data suggest that the +51 erythroid enhancer, specifically, is responsible for binding the SCL erythroid complex and also recruits PolII in erythroid cells only - this recruitment extends to +50, towards the SCL gene, and includes TAFII 250. The +53 promoter, on the other hand, is involved in recruitment of PolII and TAFII 250 in all of the cell lines with a bias towards recruitment in the opposite direction, and includes the binding of CTCF near, but not at, the promoter.

This bipartite structure was re-iterated when we examined histone H3 K4me and histone H3 K9/K14ac. All of the cell lines showed patterns for these histone marks at or close to the +53 region consistent with the activity of the promoter. Only K562 showed increased levels of histone modifications consistent with the activity of the enhancer at +51 extending to +50. We also confirmed that transcripts produced at or near the +53 element were present in all of these cell lines. Their expression, however, was at low levels relative to SCL expression (see Figure S8 for K562 data), or the expression of the β-actin gene (not shown).

## Discussion

Over the last twenty years, a number of approaches have been used to characterise regulatory elements at the SCL locus. In many of these approaches, a variety of human and murine haematopoietic cell lines have been used to identify regions capable of mediating regulatory activities on SCL [12], [14], [32], [33], [49], [52]. We describe here the first comprehensive analysis using ChIP-chip approaches and downstream analyses to facilitate the characterization of a further set of genomic cis-acting sequences which underlie the regulation of gene expression at the human SCL locus. Patterns of histone modifications, chromatin accessibility/nucleosome density and the binding of regulatory/transcriptional proteins were obtained across 256 kb of the locus in the K562 haematopoietic cell line. Based on this, six novel human regulatory elements (named −31, −13, −10, −7, +53, +57) found either upstream or downstream of SCL are characterised here, and one existing element, the SCL erythroid enhancer (+51), has been re-defined. This study effectively more than doubles the number of regulatory elements localised to the human SCL locus, and emphasises the impact that such approaches have at rapidly defining the regulatory architecture of gene loci. In addition to these, we identified a further 3 regions of interest (−26, +39, +45) by virtue of their association with peaks of histone modifications; we have not examined these in this study but they warrant further investigation. Our analysis also redefines the size of what we consider to be the SCL regulon – to a region encompassing 88 kb between the −31 element upstream of SCL and the +57 element downstream of the +51 erythroid enhancer. Both −31 and +57 bind CTCF - consistent with the role of CTCF-binding elements defining regulatory domains [35], [36]. Previous studies have suggested that the SCL regulon encompasses 65 kb [57], but this figure seems to be an underestimate given that, while it includes −31 within its boundaries, it excludes the +51 erythroid enhancer, and the +53 and +57 elements. Whilst the exact roles of +53 and +57 with respect to SCL activity have yet to be determined, we cannot exclude them from the SCL regulon until further studies are performed (see also discussion below).

The overall general regulatory features of these novel elements, and other known elements, at the SCL locus are in agreement with accepted models of regulatory function. Firstly, they are associated with histone H3 K4me, with enhancers and promoters showing biases towards H3 K4me1 and H3 K4me3 respectively. Second, they are in regions of open chromatin, characterised by low nucleosome density and FAIRE enrichments and correlate to positions of known hypersensitive sites [49]. Finally, given their open chromatin conformation, they bind transcription factors and components of the transcriptional machinery, the binding sites for some of which are conserved across other mammalian species.

Taken together with our report construct transfection data in K562, our ChIP-chip analyses identify and characterise components of the regulatory environment of the human SCL locus, some or all of which may control SCL expression. Given that our reporter assays in K562 do not necessarily recapitulate the function of these components in the correct biological environment, it will be necessary to perform further functional studies *in vivo* to determine their lineage specificity and roles in regulating SCL expression. These lines of investigation would include ChIP analysis in primary haematopoietic cells, removal (i.e., knockout) or mutation of cis-elements, or analysis of reporter transgenes under the regulatory control of these elements. Such studies have been performed for several of the known cis-elements which direct SCL expression in mouse or *Xenopus* [15], [32], [34], [57]–[61] subsequent to their identification and initial characterization in cell lines.

Upstream of SCL we identified three enhancers and one repressor element at −7, −10, −31 and −13 respectively. The element at −13 is capable of repressing luciferase expression under the control of either the SCL pro1a or SV40 promoters in erythroid (K562) and lymphoid (HPB-ALL) cells, consistent with the idea that it may regulate SCL expression when it is expressed in the erythroid lineage, and contribute to its silencing in the lymphoid lineage. It is the second repressor element identified at the SCL locus - the first being found in the 3′ UTR of the SCL gene [12]. Whilst we have not confirmed this here because of a lack of an antibody for ETV6/7 which works well in ChIP, the ETV6/7 (TEL1/TEL2) proteins are still likely candidates to mediate the activity of this repressor element. The −10 region is capable of enhancing luciferase expression under the control of both the SCL pro1a and SV40 promoters in human erythroid cells and the endogenous −10 binds a number of transcription factors including GATA1, E2A and LDB1 *in vivo*. A murine genomic sequence equivalent to −10 has been shown to enhance expression of murine SCL in erythroid cells [33]. The −7 element also has enhancer activity in reporter assays in K562 and binds GATA1 *in vivo*. However, whilst −7 enhanced luciferase expression under the control of the SV40 promoter, it was not capable of enhancing reporter expression under the control of the SCL pro1a, suggesting that the local chromatin environment may be important in regulating its activity *in vivo* or that it regulates SCL activity but not through enhancement of SCL pro1a. Furthermore, the fact that both −10 and −7 may also be active in the lymphoid lineage (by virtue of their activity in HPB-ALL cells) also suggests that, unlike +51 erythroid enhancer, their regulation of SCL is not lineage-specific. Conversely, the −31 element was able to enhance luciferase expression under the control of SCL pro1a, but not under the control of the SV40 promoter, suggesting that whilst the chromatin environment may not be important for its activity, its role as an enhancer has additional specificity for SCL pro1a.

The −7 and −31 enhancers both bind CTCF *in vivo* in erythroid cells and, in the case of the latter, CTCF binding is highly enriched in ChIP-chip. Spatially, the binding of CTCF at these enhancers suggests that its role is not related to insulator activity at these sites, but is that of a transcription factor [37]. However, the binding pattern of CTCF at −31 is not defined by a single peak of enrichment in ChIP-chip - it is likely to bind to several sites over at least 2 kb interval centered at −31. This suggests that its role at this element may be complex and may suggest that other elements are juxtaposed nearby. This model, and the fact that no other regulatory sequences for SCL have been identified distal to −31 (towards SIL), would be consistent with the binding of CTCF at −31 or nearby sequences defining an insulator boundary between SCL regulatory elements and those of the neighbouring SIL gene. Enhancer blocking assays confirmed that −31 does indeed have insulator activity and supports our interpretation of −31 as defining the SIL/SCL regulatory boundary.

We also detected a novel region which binds CTCF downstream of SCL at +57. Unlike −31, this region does not demonstrate enhancer activity in reporter assays, but does have insulator enhancer-blocking activity. The location of +57, which coincides with a boundary between high levels of H3 K27me3 on one side and H3 K4me1,2 on the other in K562, is highly suggestive of a role as an insulator also with barrier activity. An insulator at this location would be required for the compartmentalisation of elements involved in SCL regulation (including the +51 region six kb away) from those of CYP4A22 as well as preventing the spread of repressive histone marks into the SCL regulatory domain. However, given that +51 is located between the MAP17 gene and an active bi-directional promoter at +53, the possibility that CTCF may mediate other insulator functions to compartmentalise the regulation of these genes, would also be necessary. The binding of CTCF near +53 and upstream of the MAP17 promoter at/near the recently identified MAP17 enhancer[31], both of which have insulator enhancer-blocking activity, support this hypothesis and underlines the complexity of the compartmentalisation required to delineate the SCL regulatory domain.

This complexity is no better exemplified than by the region surrounding the erythroid enhancer at +51 (Figure S9). The murine equivalent of this enhancer (SCL +40 in mouse), has been shown to regulate SCL expression during primitive and definitive erythropoiesis and in the midbrain [15]. Furthermore, the histone modification profiles and TF binding of murine +40 and human +51 [14], [15] are in agreement with the results we described in K562 and in cell types which do not express SCL. In human, the +51 enhancer is flanked on one side by a bi-directional promoter at +53, which is associated with transcripts of no known function. These short transcripts (<500 bp) are spliced but have no substantial open-reading frames, suggesting they may be non-coding RNAs [62].

The erythroid enhancer is highly active in K562 cells at enhancing reporter expression under the control of the SCL pro1a and SV40 promoter. Along with the +53 promoter, +51 recruits PolII, although the recruitment to the enhancer element is distinguishable from that to the +53. Mechanistically, this suggests that the enhancer may be involved in the delivery of PolII and TAFII 250 to the SCL promoters, but equally it could be involved in recruitment of the transcriptional machinery to +53, as the exact relationship between these two elements is not yet known. Furthermore, whilst the roles of any ncRNAs originating from +53 are not known, these may involve the maintenance of an open chromatin status (in addition to the presumptive insulator role of the +57 element) at one boundary of the SCL regulatory domain, or transcriptional interference regulating the +51 enhancer [63].

A further complexity of this region is that activating histone modifications, TAFII 250 and PolII are also present at +50, flanking the core erythroid enhancer, which are distinguishable from peaks of enrichment at +51. Recent studies of the murine SCL stem cell enhancer (+20 in human) have shown that additional sequences flanking it specifically boost SCL activity *in vivo* [64]. Transient reporter assays containing the murine equivalents of both +50 and +51 also showed increased enhancer activity compared with constructs lacking +50 [15]. Whether +50 represents an additional regulatory element or a “booster” element is not clear at this time.

The +51 erythroid enhancer also binds the SCL-containing erythroid complex (SEC) in K562 cells. We have also confirmed the binding of the SEC in another human erythroid cell line (HEL 92.1.7). These data point to the possibility that SCL regulates its own expression, however, SEC binding in primary primitive erythroblasts would be required to confirm SCL auto-regulation *in vivo.* To date, there is evidence from analysis of the murine SCL erythroid enhancer to support this hypothesis [15]. In HSCs, activity of the SCL stem cell enhancer (+20/+21 in human) is independent of SCL expression [59]. Our data are therefore consistent with a model where once SCL expression has been established in HSCs, maintenance of SCL expression during differentiation towards erythroid cells is at least partly achieved through an auto-regulatory feedback loop acting through the +51 erythroid enhancer and mediated by the binding of the SCL-containing erythroid complex. We are currently exploring several avenues of investigation to further understand the function of this enhancer and the ever-increasing complexities of SCL transcriptional regulation.

## Materials and Methods

### SCL Tiling Array Construction

Oligonucleotide primers pairs (Table S2) were designed from genomic sequence and used to prepare amplicons and arrays as described elsewhere [19].

### Chromatin Immunoprecipitation and FAIRE

Human cell line K562 [65] was cultured in DMEM, 9% fetal calf serum, 1% penicillin-streptomycin and 2 mM L-glutamine. Human cell lines HEL 92.1.7 [66], U937 [67], HL-60 [68], and HPB-ALL [69] were cultured in RPMI 1640, fetal calf serum (18% for U937, 9% for HEL 92.1.7, HL-60 and HPB-ALL), 1% penicillin streptomycin and 2 mM L-glutamine. ChIP was performed as described elsewhere [70]. ChIP antibodies are described in Table S3. Ten micrograms of antibody was used in each ChIP assay. Formaldehyde-based chromatin fractionation assays (FAIRE) [47], [48] were performed using cross-linked chromatin prepared as for ChIP.

### Fluorescent DNA Labelling, Microarray Hybridization and Quantitation

Fluorescently-labelled DNA samples were prepared from unamplified input/ChIP/FAIRE DNAs and hybridized to microarrays for 45 hours using an automated hybridization station (HS 4800™, TECAN). Microarrays were scanned using a ScanArray 4000 XL (Perkin Elmer). Mean spot intensities from images were quantified using ProScanArray® Express (Perkin-Elmer) with background subtraction. Mean ratios and standard deviations (SDs) for all array elements spotted in triplicate were calculated and the datasets normalized to the median ratio per hybridization. Final datasets for each assay were derived from the mean values of three independent biological replicate experiments. Transcription factor, RNA polymerase II (PolII) and TAFII 250 ChIP-chip experiments were normalised with the relevant antisera control datasets. Significant enrichments in normalised transcription factor ChIP-chip experiments were considered to be those values that were more than 4 standard deviations away from the mean ratio of background levels. Background levels were derived from genomic regions represented as SCL tiling arrays which did not contain known non-coding regulatory sequences at the time of analyis (chromosome 1 genomic co-ordinates 47262288–47343557 and 47424426–47489322 (NCBI build 35). FAIRE, histone modification and histone H3/H2B enrichments/depletions were interpreted based on visualizations of data plots.

### Reporter Assays

Putative and known regulatory elements were tested in replicate experiments for their ability to promote, enhance or repress the expression of the firefly luciferase gene in pGL3 reporter construct (Promega). For promoter assays, PCR products (Table S4) were cloned upstream of the firefly luciferase gene into the *Hin*dIII and *Kpn*I cloning sites of the promoterless pGL3 basic vector. For enhancer/repressor assays, PCR products (Table S4) were cloned in the *Bam*HI cloning site downstream of the firefly luciferase gene under the control of either the SV40 promoter or SCL promoter 1a. Aliquots of 5×10^6^ K562 cells were transferred to Gene Pulsar 0.2 cm cuvettes (Bio-Rad) and transfected with 4 µg of luciferase reporter construct and 1 µg of a control plasmid expressing β-galactosidase using nucleofection (Amaxa, protocol T-016) and cultured for 48 hours. Cell lysates were prepared with 100 µL of 1X lysis buffer (Promega). 20 µL of cell lysates were used for luminescent β-galactosidase assays (Clontech) and luciferase assays (Promega) using a luminometer (LB 930, Berthold). Luciferase activities were then normalised with the corresponding β-galactosidase values and expressed relative to the relevant negative control experiment.

For enhancer-blocking assays, PCR products (Table S5) spanning CTCF binding sites were cloned into the *AscI* site of the vector pNI [71], [72]. Each construct was linearised with *SalI* and 1 µg was transfected (Amaxa, protocol T-016) into 1×10^7^ K562 cells. After 24 hr of recovery, cells were plated on soft agar with geneticin (G418) (750 µg/ml). G418 resistant K562 colonies were counted 2.5–3 weeks after selection and compared to results obtained with pNI (“insulator-less” vector) and pNI-CD (containing 2 copies of the 250 bp chicken b-globin core HS4 insulator).

### Quantitative Real-Time PCR of ChIP DNAs and cDNA

K562 and HEL 92.1.7 ChIP and input DNAs were quantified for genomic DNA sequences represented on the human SCL tile path array. SyBr green PCR (Applied Biosystems) was performed on a 7700 sequence detection system (Applied Biosystems). The oligonucleotide primer pairs used are found in Tables S6 and S7 respectively. Each quantitative real-time PCR assay was performed in triplicate. Fold enrichments were calculated as described previously [14]. The mean quantitative PCR values were normalised against the corresponding array data by deriving the median ratio of both datasets and scaling the values accordingly.

Total cellular RNA from human cell lines was purified using TRIZOL reagent (Invitrogen) (www.sanger.ac.uk/Projects/Microarrays/arraylab/methods.shtml). Single-stranded cDNA from these RNAs was synthesized from 1 µg total RNA using SuperScript™ II RNase H^−^ reverse transcriptase according to manufacturer's instructions (Invitrogen). These cDNA were then used to quantify the level of novel transcripts, SCL and the β-actin control. The oligonucleotide primer pairs used are found in Table S8. SyBr green PCR (Applied Biosystems) was performed on a 7700 sequence detection system (Applied Biosystems). C_t_ values were extracted and ΔC_t_ values of transcripts a, b and c calculated relative to the level of SCL and/or β-actin control.

### Computational Analyses

Hierarchical clustering was performed using Cluster 3.0 [73] (Euclidean distance similarity metric with average linkage) and visualised with Java Treeview [74]. Comparative sequence alignments of novel regulatory regions found in human, chimp, mouse, rat and dog were obtained from the UCSC Genome Browser (http://genome.ucsc.edu/). Transcription factor binding sites were identified using the TFSEARCH (http://www.cbrc.jp/research/db/TFSEARCH.html) [75], [76] and TESS (http://www.cbil.upenn.edu/tess/) [77] web servers. Five-way regulatory potential scores [53], [54] were visualized on the UCSC Genome Browser.

## Supporting Information

Figure S1 Reproducibility of ChIP-chip and related experiments using the human SCL genomic tiling array. The figure shows profiles generated from three independent biological replicate experiments (shown as red, green and yellow joined-up lines) for each of four different assays: histone H3 K9/K14ac (panel a), GATA1 (panel b), FAIRE (panel c) and histone H3 (panel d). In each panel, the x-axis is the genomic sequence co-ordinate (NCBI build 35) and the y-axis is the enrichment obtained in ChIP-chip assays expressed in log2 scale. Schematic diagram at the bottom of the figure shows the genomic organisation of SCL and its neighbouring genes. Exons are shown as vertical blocks with gene names and direction of transcription shown above. Transcripts denoted by a,b and c refer to transcripts of unknown function (see also text). Vertical lines at the bottom (with dotted lines through all the panels) show the location of known and novel regulatory regions at the SCL locus. Promoters are denoted by P. Other nomenclature refers to the distance in kb from SCL promoter 1a. We assessed the performance of every array element across multiple independent experiments; the mean coefficient of variance (cv) in the ratios reported by array elements ranged between 7–13% for all of the assays described in this paper.
(0.90 MB TIF)

Figure S2 Comparison between enrichments obtained across the human SCL locus by ChIP-chip with those obtained from real-time SyBr Green PCR analysis of ChIP samples. (A) Histone H3 K9/K14ac in K562. (B) GATA1 in K562. Fold enrichments in log2 scale are shown on the y-axis and datapoints across the locus are shown on the x-axis for each histogram. Enrichments reported by the array (grey bars) and those reported by real-time PCR (black bars) are shown as pairs for each amplicon tested. Data in panel a are ordered with respect to their genomic co-ordinates and bracketed according to their location across the human SCL locus. Data in panel b are ordered with respect to their level of ChIP enrichments across the human SCL locus. See also Tables S6 and S7 for genomic co-ordinates. The nomenclature of data points refers to the distance in kb that the amplicon is located upstream (−) or downstream (+) from the promoter of the closest gene. NC  =  negative control regions.
(0.76 MB TIF)

Figure S3 Profiles of binding for members of the SCL erythroid transcription factor complex across the human SCL locus in the K562 cell line. The transcription factors studied are named at the left of each panel. E47 and E12 are isoforms of E2A. Dots on the joined-up lines represent the data obtained for each genomic tiling array element. In each panel, the x-axis is the genomic sequence co-ordinate (NCBI build 35) and the y-axis is the enrichment obtained in ChIP-chip assays expressed in log2 scale. Schematic diagram at the bottom of the figure shows the genomic organisation of SCL and its neighbouring genes. Exons are shown as vertical blocks with gene names and direction of transcription shown above. Transcripts denoted by a, b and c refer to transcripts of unknown function. Vertical lines at the bottom (with dotted lines through all the panels) show the location of known and novel regulatory regions at the SCL locus. Promoters are denoted by P. Other nomenclature refers to the distance in kb from SCL promoter 1a.
(0.96 MB TIF)

Figure S4 Correlation of nucleosome density and chromatin fractionation (FAIRE) assay across the human SCL locus. Datapoints for each array tile are plotted as a function of chromatin fractionation/FAIRE (y-axis) and nucleosome density (x-axis). All data are plotted as log2 values. Nucleosome densities are derived as the mean value obtained from ChIP-chip analysis of histone H3 and H2B. A strong negative correlation between nucleosome density and chromatin fractionation was obtained with a correlation co-efficient of R  = -0.861.
(0.52 MB TIF)

Figure S5 Conserved transcription factor binding sites found at the novel -13 regulatory region. Sequence alignments are shown for human, chimp, mouse, rat and dog. Genomic sequence co-ordinates for each region of homology are shown in brackets (taken from their respective genome builds). Bases of sequence identity are denoted with an asterisk (*). Site for ETV6/7 (TEL1/2) is boxed in bold. Sites are shown (boxed) for a variety of other transcription factors including Sp1, PEA3, ETS-1, GR (glucocorticoid receptors), RAR-x (retinoic acid receptors), AP1 (activator proteins), and NFAT-x (nuclear factors of activated T cells).
(0.65 MB TIF)

Figure S6 Conserved transcription factor binding sites found at the human SCL +51 erythroid enhancer. Sequence alignments are shown for human, chimp, mouse, rat and dog. Genomic sequence co-ordinates for each region of homology are shown in brackets (taken from their respective genome builds). Bases of sequence identity are denoted with an asterisk (*). Sites for GATA1 (and other family members), the SCL erythroid transcriptional complex are boxed in bold. Sites are shown (boxed) for a variety of other transcription factors including Sp1, E12, MyoD (E-box), RAR-x (retinoic acid receptors), and AP-1,2,4 (activator proteins).
(0.74 MB TIF)

Figure S7 Binding of members of the SCL erythroid complex to the +51 region in the erythroid HEL 92.1.7 cell line. Histogram shows the ChIP enrichments obtained for GATA1, E2A (E12 and E47), SCL, LDB1 and LMO2 at +51 (black bars). Grey bars show the value equal to two standard deviations above the mean ChIP enrichments for a series of negative control regions (NCi - Ncxi as in Figure S2a) across the SCL locus. Primer pairs for these negative control regions and +51 are shown in Tables S6 and S7.
(0.60 MB TIF)

Figure S8 Expression of transcripts of unknown function at the +53 region in the K562 cell line. Histogram shows real-time SyBr green quantitative PCR results of transcripts a, b and c expressed in log10 scale relative to the level of expression of SCL (SCL is assigned an arbitrary level of expression). PCR amplifications from samples which were reverse transcribed into cDNA are shown as black bars. Amplicon for transcripts a and b was from coding sequence shared by both transcripts - thus expression results for these are presented collectively as a/b. Negative controls for PCR amplification in the absence of reverse transcription are shown as grey bars.
(0.55 MB TIF)

Figure S9 Schematic diagram of the biological events identified at the human SCL erythroid enhancer in the K562 cell line. At the top of the figure, DNA is shown as the black line wrapped around nucleosomes (blue/grey spheres). Regions of accessible chromatin are shown as regions with fewer nucleosomes per unit length of DNA. Location of transcription factors, RNApolII and TAFII 250 are shown by larger grey and black spheres and ovoids. Histone modifications are shown as flags on the nucleosomes and are histone H3 K9/K14ac (Ac), histone H3 K4me3 (3 M), histone H3 K4me2 (2 M), and histone H3 K4me1 (1M). The four kb block of genomic DNA (+50 to +53) shown at the bottom half of the figure is numbered in one kb intervals according to distance in kb from the SCL pro1a. Genomic sequence co-ordinates are from NCBI build 35. The core erythroid enhancer at +51 is shown as the black horizontal bar and the extent of the bar defines the region which drives enhancer activity in transient reporter assays (see Figure 4). The bidirectional promoter at +53 is shown as the black horizontal bar with arrowheads and the bar extent defines the region which has bidirectional promoter activity in transient reporter assays.
(1.26 MB TIF)

Table S1 Array elements which show significant enrichments for each member of the SCL erythroid complex in K562 cells. The names of array elements shown in the third column are as described in Table S2. Significant enrichments derived as the mean ratio from multiple experiments are shown in log2 scale in the second column. Genomic sequence co-ordinates are from NCBI build 35.
(0.09 MB DOC)

Table S2 Oligonucleotide primer pairs used to PCR amplify array elements for the human SCL tiling array. Amplicon names used in our laboratory are shown in the first column. Amplicon sizes and genomic sequence co-ordinates are from NCBI build 35.
(0.60 MB DOC)

Table S3 Antibodies used for ChIP-chip assays performed in this study. Antibodies for histone modifications, transcription factors, histones and antisera controls are listed along with their supplier and catalogue numbers.
(0.05 MB DOC)

Table S4 Oligonucleotide primer pairs used to PCR amplify the regions cloned into the pGL3 luciferase reporter constructs. Construct names in the first column are as described in Figure 4. The BamHI, Kpn1 and HindIII sites added to the sequence of each primer are shown in brackets. Amplicon sizes and genomic sequence co-ordinates are from NCBI build 35.
(0.03 MB DOC)

Table S5 Oligonucleotide primer pairs used to PCR amplify the regions cloned into the enhancer blocking reporter constructs. Construct names in the first column are as described in Figure 4. The AscI sites added to the sequence of each primer are shown in brackets. Amplicon sizes and genomic sequence co-ordinates are from NCBI build 35.
(0.03 MB DOC)

Table S6 Oligonucleotide primer pairs used to perform SyBr green quantitative real-time PCR for regions enriched for histone H3 K9/K14ac. Amplicon names in the first column are as shown in Figure S2 (panel a) while the second column lists alternative names which describe corresponding amplicons found in Table S2. Amplicon sizes and genomic sequence co-ordinates are taken from NCBI build 35.
(0.12 MB DOC)

Table S7 Oligonucleotide primer pairs used to perform SyBr green quantitative real-time PCR for regions enriched for GATA1. Amplicon names in the first column are as described in Figure S2 (panel b) while the second column lists alternative names which describe corresponding amplicons found in Table S2. Amplicon sizes and genomic sequence co-ordinates are from NCBI build 35.
(0.04 MB DOC)

Table S8 Oligonucleotide primer pairs used to perform SyBr green quantitative real-time PCR to detect reverse-transcribed cDNA for transcripts a, b and c of unknown function. Amplicon for transcripts a and b was from coding sequence shared by both transcripts - thus one amplicon represents both transcripts. The primer pairs for SCL and β-actin are also shown. Alternative names for the primer pairs are shown. Amplicon sizes and genomic sequence co-ordinates are taken from NCBI build 35. n.a.  =  not applicable.
(0.03 MB DOC)
