# Supplementary figures and images for: Genomic Approaches Uncover Increasing Complexities in the Regulatory Landscape at the Human SCL (TAL1) Locus
Source: PLoS One. 2010 Feb 5;5(2):e9059. doi: 10.1371/journal.pone.0009059 (PMC2816701; doi:10.1371/journal.pone.0009059)

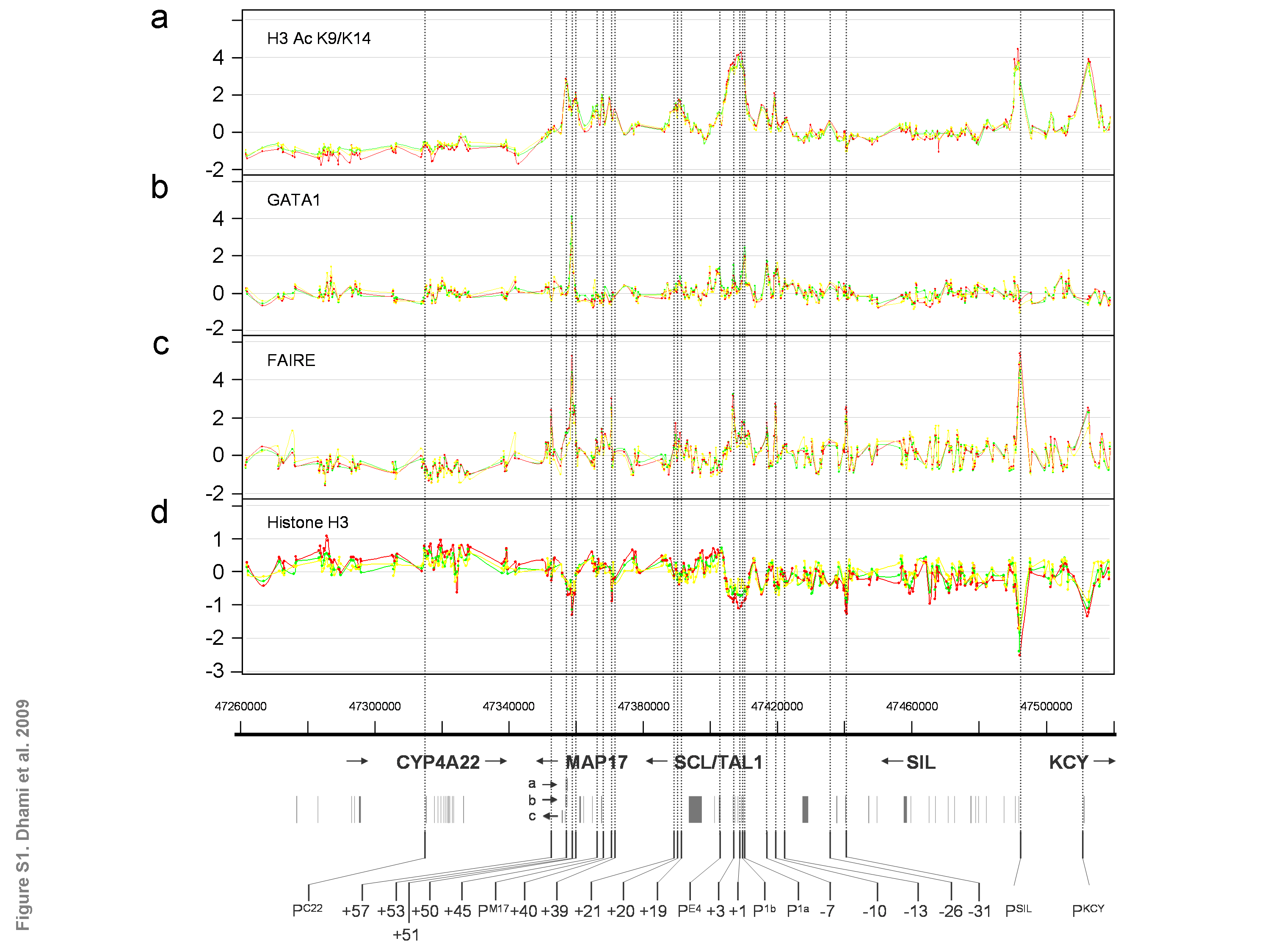

Supplement: Figure S1 — Reproducibility of ChIP-chip and related experiments using the human SCL genomic tiling array. The figure shows profiles generated from three independent biological replicate experiments (shown as red, green and yellow joined-up lines) for each of four different assays: histone H3 K9/K14ac (panel a), GATA1 (panel b), FAIRE (panel c) and histone H3 (panel d). In each panel, the x-axis is the genomic sequence co-ordinate (NCBI build 35) and the y-axis is the enrichment obtained in ChIP-chip assays expressed in log2 scale. Schematic diagram at the bottom of the figure shows the genomic organisation of SCL and its neighbouring genes. Exons are shown as vertical blocks with gene names and direction of transcription shown above. Transcripts denoted by a,b and c refer to transcripts of unknown function (see also text). Vertical lines at the bottom (with dotted lines through all the panels) show the location of known and novel regulatory regions at the SCL locus. Promoters are denoted by P. Other nomenclature refers to the distance in kb from SCL promoter 1a. We assessed the performance of every array element across multiple independent experiments; the mean coefficient of variance (cv) in the ratios reported by array elements ranged between 7–13% for all of the assays described in this paper. (0.90 MB TIF) [file pone.0009059.s001.tif]

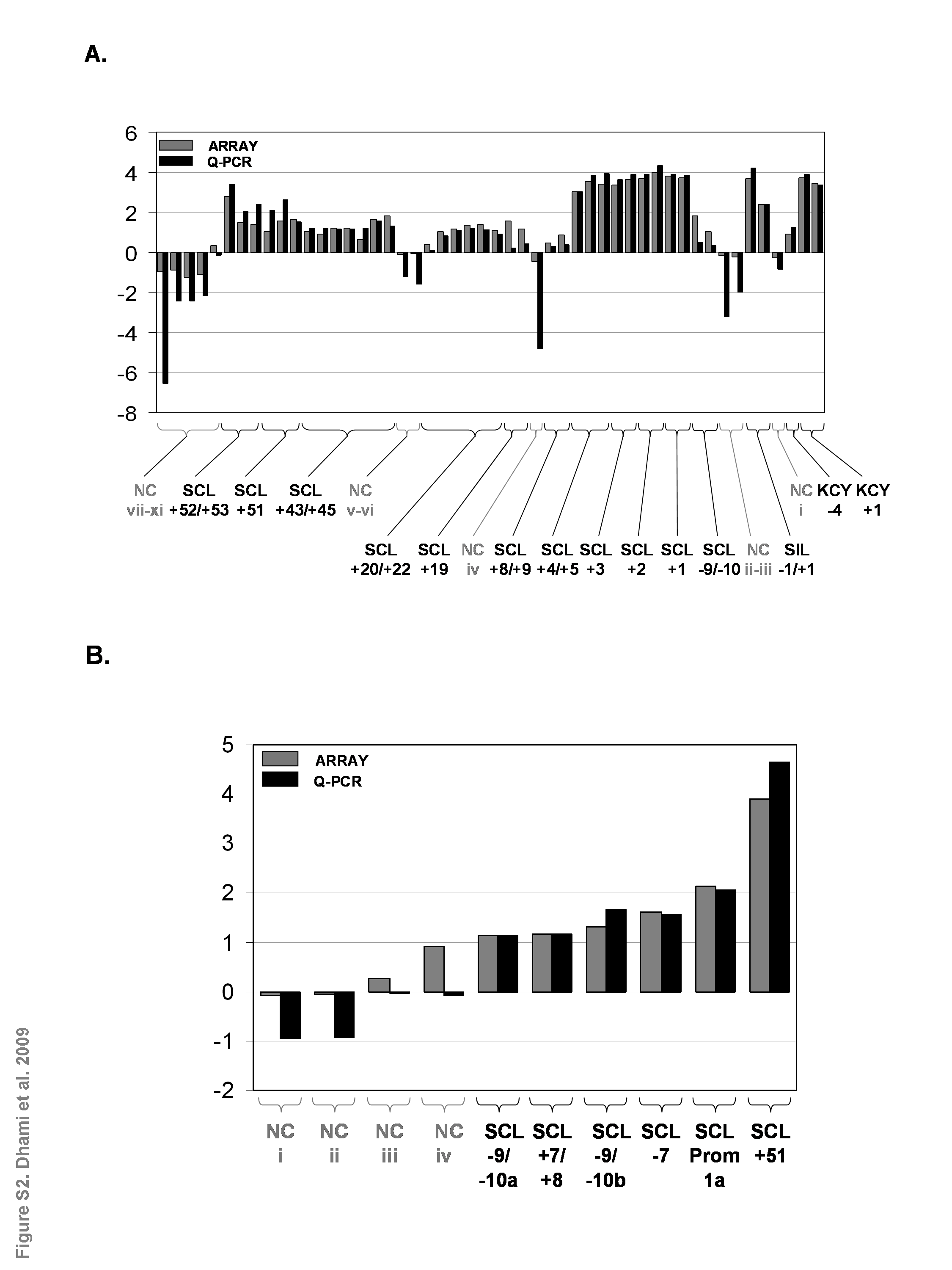

Supplement: Figure S2 — Comparison between enrichments obtained across the human SCL locus by ChIP-chip with those obtained from real-time SyBr Green PCR analysis of ChIP samples. (A) Histone H3 K9/K14ac in K562. (B) GATA1 in K562. Fold enrichments in log2 scale are shown on the y-axis and datapoints across the locus are shown on the x-axis for each histogram. Enrichments reported by the array (grey bars) and those reported by real-time PCR (black bars) are shown as pairs for each amplicon tested. Data in panel a are ordered with respect to their genomic co-ordinates and bracketed according to their location across the human SCL locus. Data in panel b are ordered with respect to their level of ChIP enrichments across the human SCL locus. See also Tables S6 and S7 for genomic co-ordinates. The nomenclature of data points refers to the distance in kb that the amplicon is located upstream (−) or downstream (+) from the promoter of the closest gene. NC = negative control regions. (0.76 MB TIF) [file pone.0009059.s002.tif]

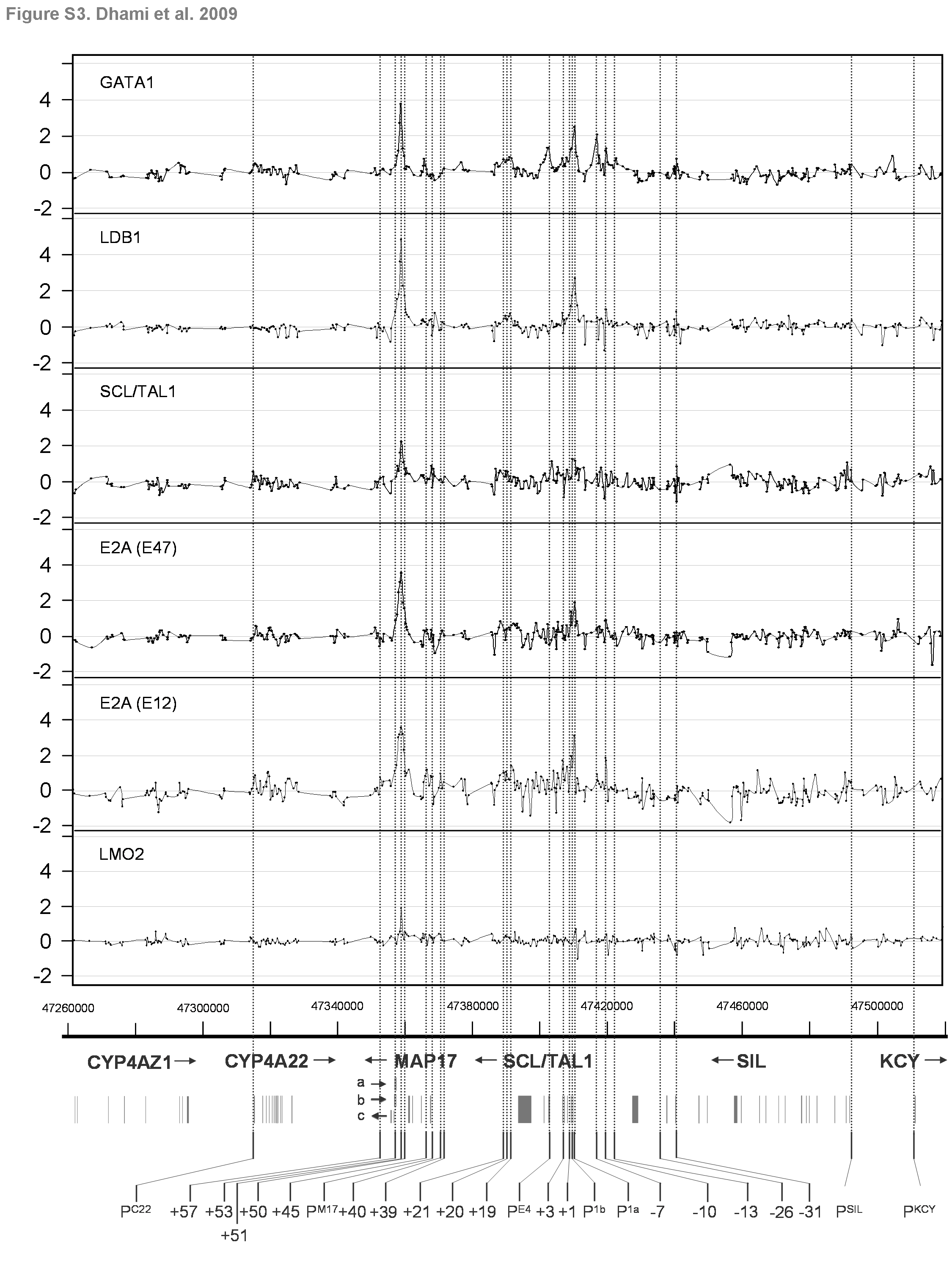

Supplement: Figure S3 — Profiles of binding for members of the SCL erythroid transcription factor complex across the human SCL locus in the K562 cell line. The transcription factors studied are named at the left of each panel. E47 and E12 are isoforms of E2A. Dots on the joined-up lines represent the data obtained for each genomic tiling array element. In each panel, the x-axis is the genomic sequence co-ordinate (NCBI build 35) and the y-axis is the enrichment obtained in ChIP-chip assays expressed in log2 scale. Schematic diagram at the bottom of the figure shows the genomic organisation of SCL and its neighbouring genes. Exons are shown as vertical blocks with gene names and direction of transcription shown above. Transcripts denoted by a, b and c refer to transcripts of unknown function. Vertical lines at the bottom (with dotted lines through all the panels) show the location of known and novel regulatory regions at the SCL locus. Promoters are denoted by P. Other nomenclature refers to the distance in kb from SCL promoter 1a. (0.96 MB TIF) [file pone.0009059.s003.tif]

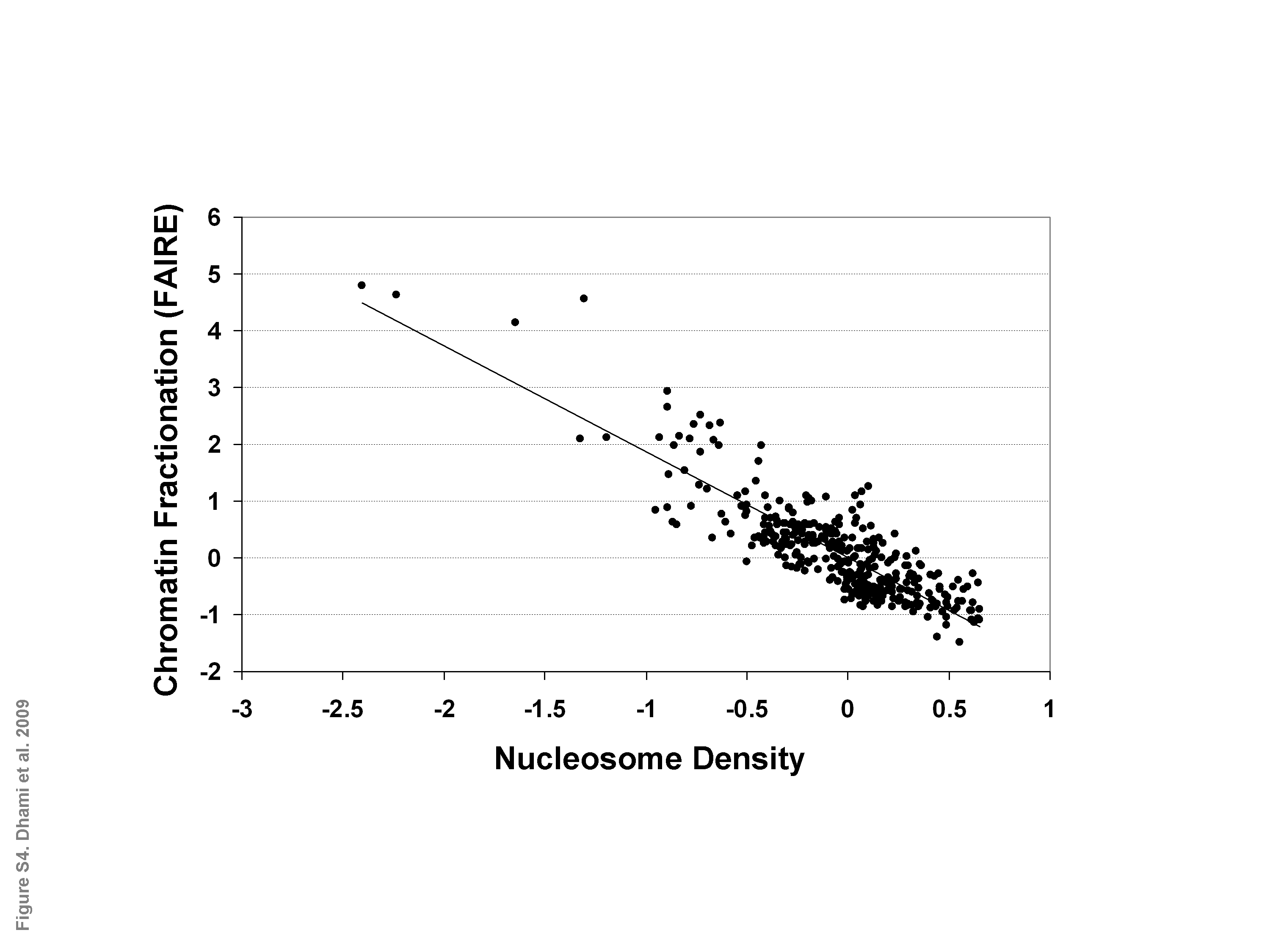

Supplement: Figure S4 — Correlation of nucleosome density and chromatin fractionation (FAIRE) assay across the human SCL locus. Datapoints for each array tile are plotted as a function of chromatin fractionation/FAIRE (y-axis) and nucleosome density (x-axis). All data are plotted as log2 values. Nucleosome densities are derived as the mean value obtained from ChIP-chip analysis of histone H3 and H2B. A strong negative correlation between nucleosome density and chromatin fractionation was obtained with a correlation co-efficient of R = -0.861. (0.52 MB TIF) [file pone.0009059.s004.tif]

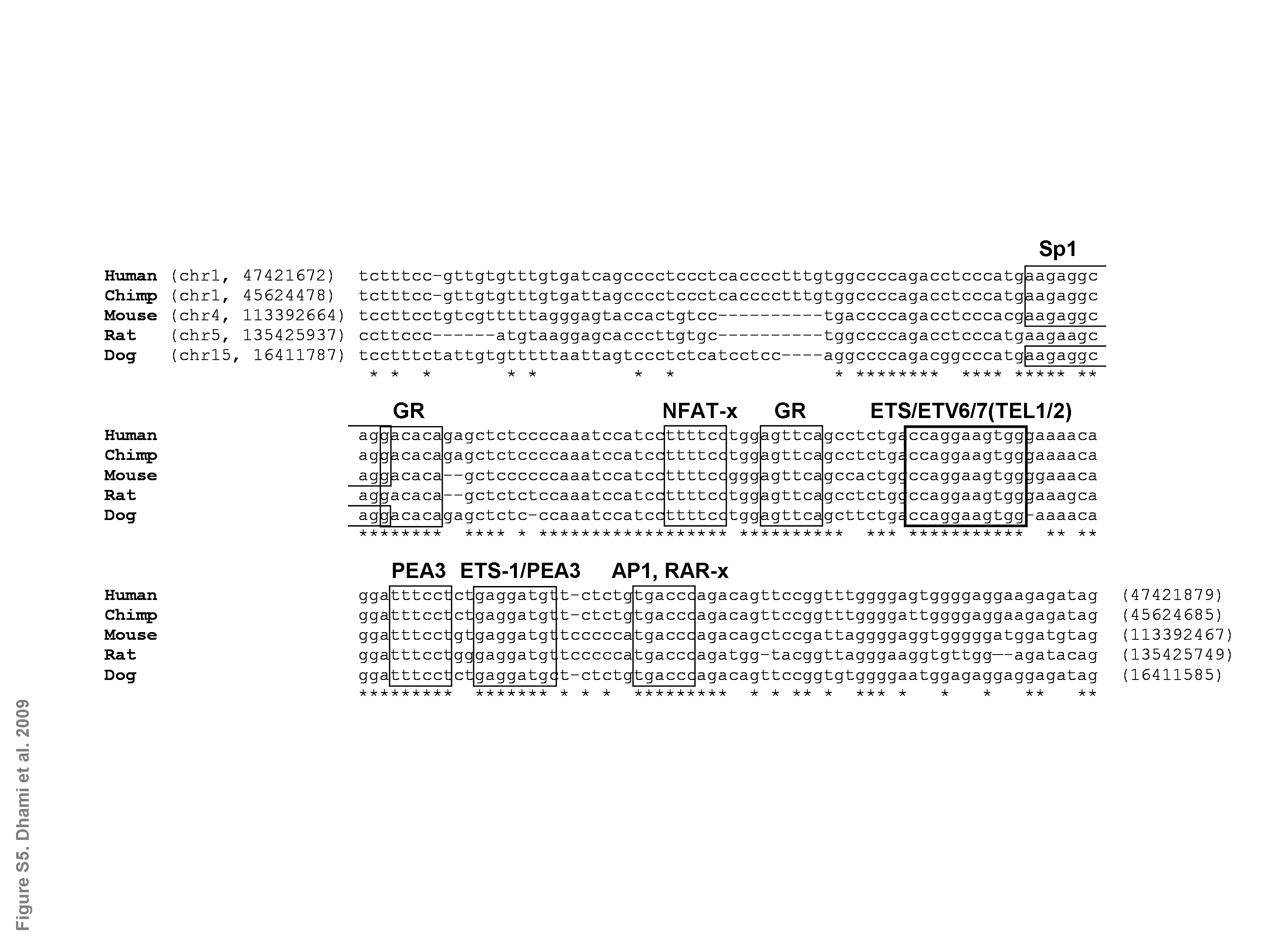

Supplement: Figure S5 — Conserved transcription factor binding sites found at the novel -13 regulatory region. Sequence alignments are shown for human, chimp, mouse, rat and dog. Genomic sequence co-ordinates for each region of homology are shown in brackets (taken from their respective genome builds). Bases of sequence identity are denoted with an asterisk (*). Site for ETV6/7 (TEL1/2) is boxed in bold. Sites are shown (boxed) for a variety of other transcription factors including Sp1, PEA3, ETS-1, GR (glucocorticoid receptors), RAR-x (retinoic acid receptors), AP1 (activator proteins), and NFAT-x (nuclear factors of activated T cells). (0.65 MB TIF) [file pone.0009059.s005.tif]

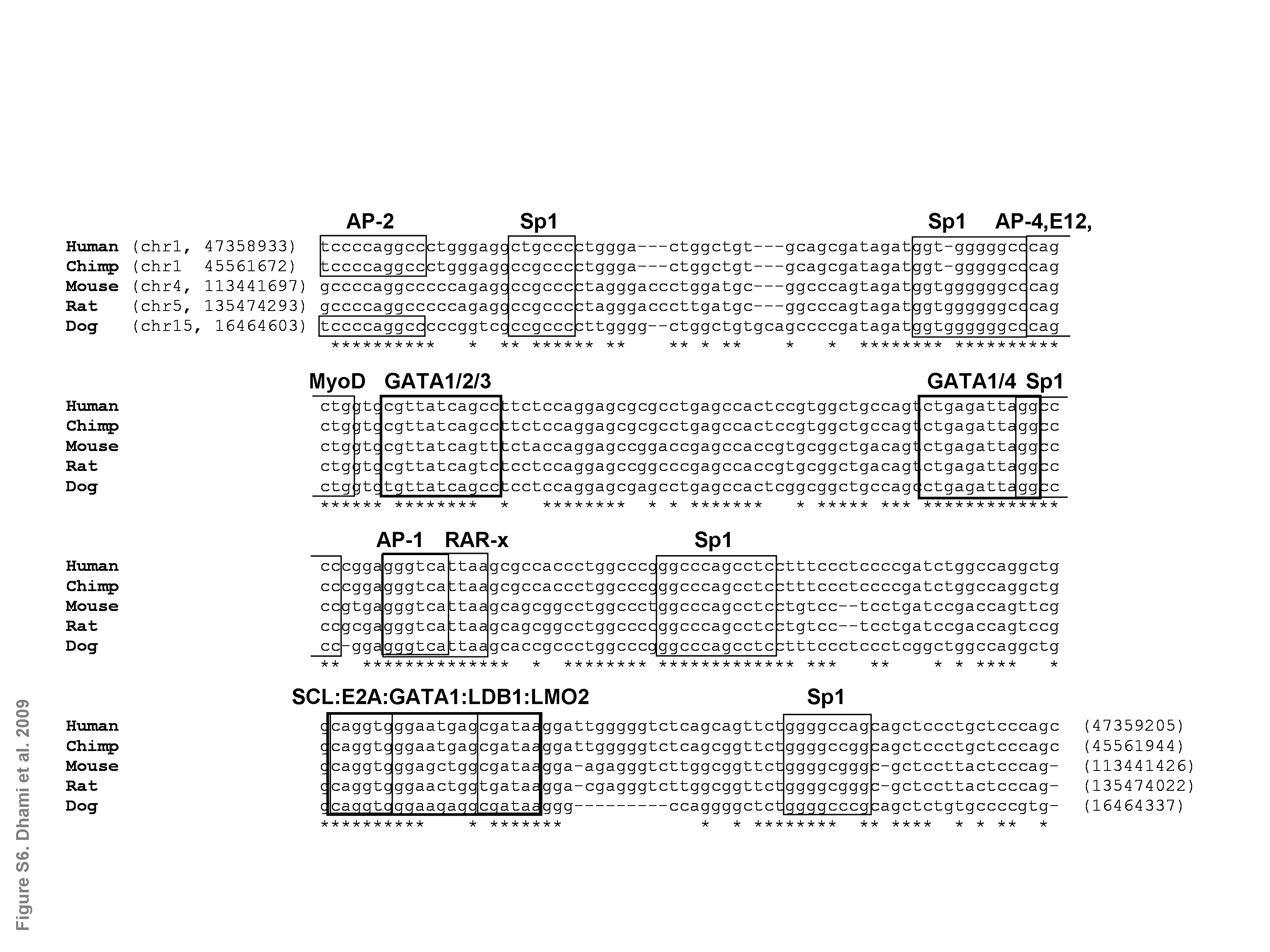

Supplement: Figure S6 — Conserved transcription factor binding sites found at the human SCL +51 erythroid enhancer. Sequence alignments are shown for human, chimp, mouse, rat and dog. Genomic sequence co-ordinates for each region of homology are shown in brackets (taken from their respective genome builds). Bases of sequence identity are denoted with an asterisk (*). Sites for GATA1 (and other family members), the SCL erythroid transcriptional complex are boxed in bold. Sites are shown (boxed) for a variety of other transcription factors including Sp1, E12, MyoD (E-box), RAR-x (retinoic acid receptors), and AP-1,2,4 (activator proteins). (0.74 MB TIF) [file pone.0009059.s006.tif]

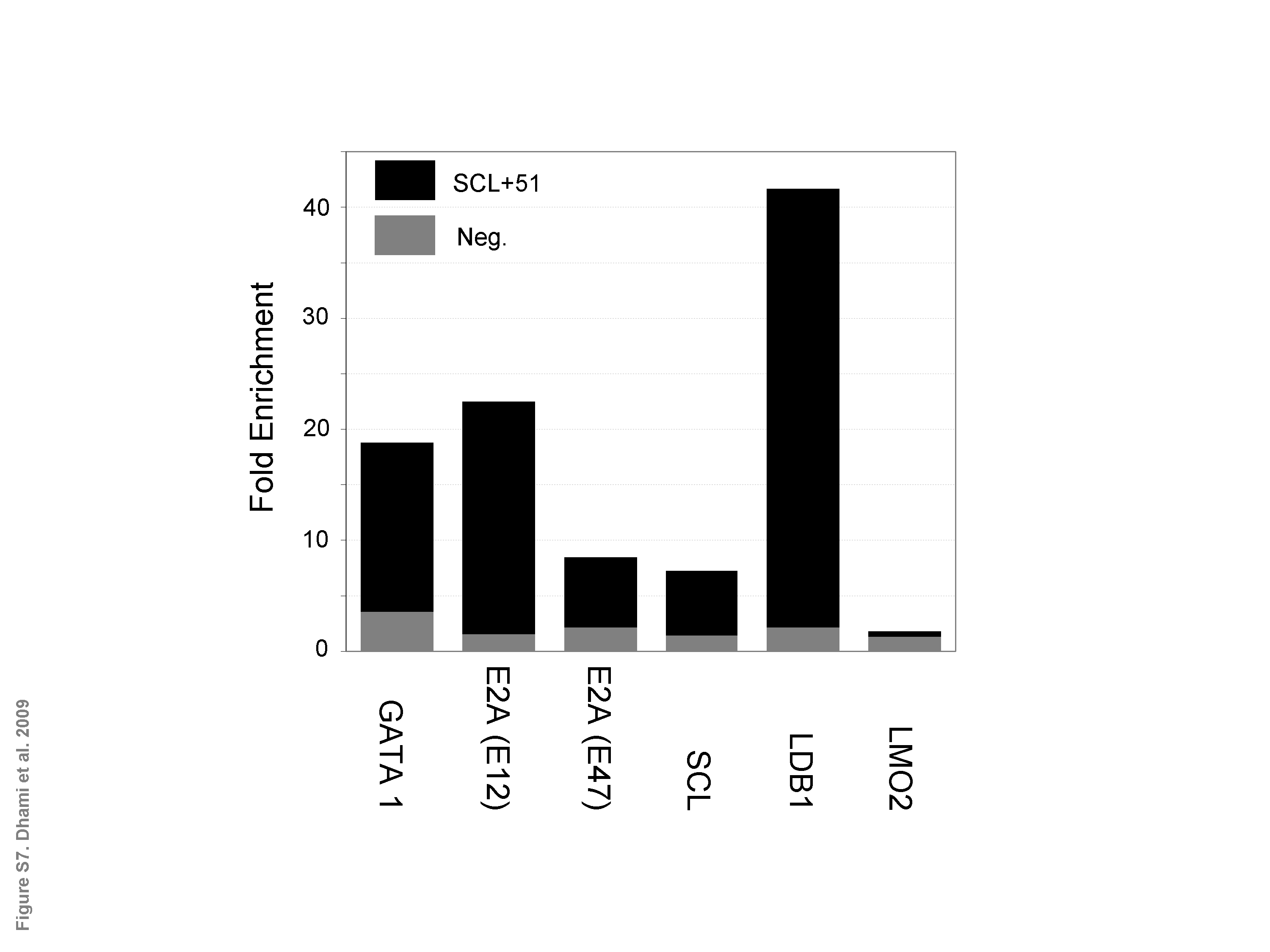

Supplement: Figure S7 — Binding of members of the SCL erythroid complex to the +51 region in the erythroid HEL 92.1.7 cell line. Histogram shows the ChIP enrichments obtained for GATA1, E2A (E12 and E47), SCL, LDB1 and LMO2 at +51 (black bars). Grey bars show the value equal to two standard deviations above the mean ChIP enrichments for a series of negative control regions (NCi - Ncxi as in Figure S2a) across the SCL locus. Primer pairs for these negative control regions and +51 are shown in Tables S6 and S7. (0.60 MB TIF) [file pone.0009059.s007.tif]

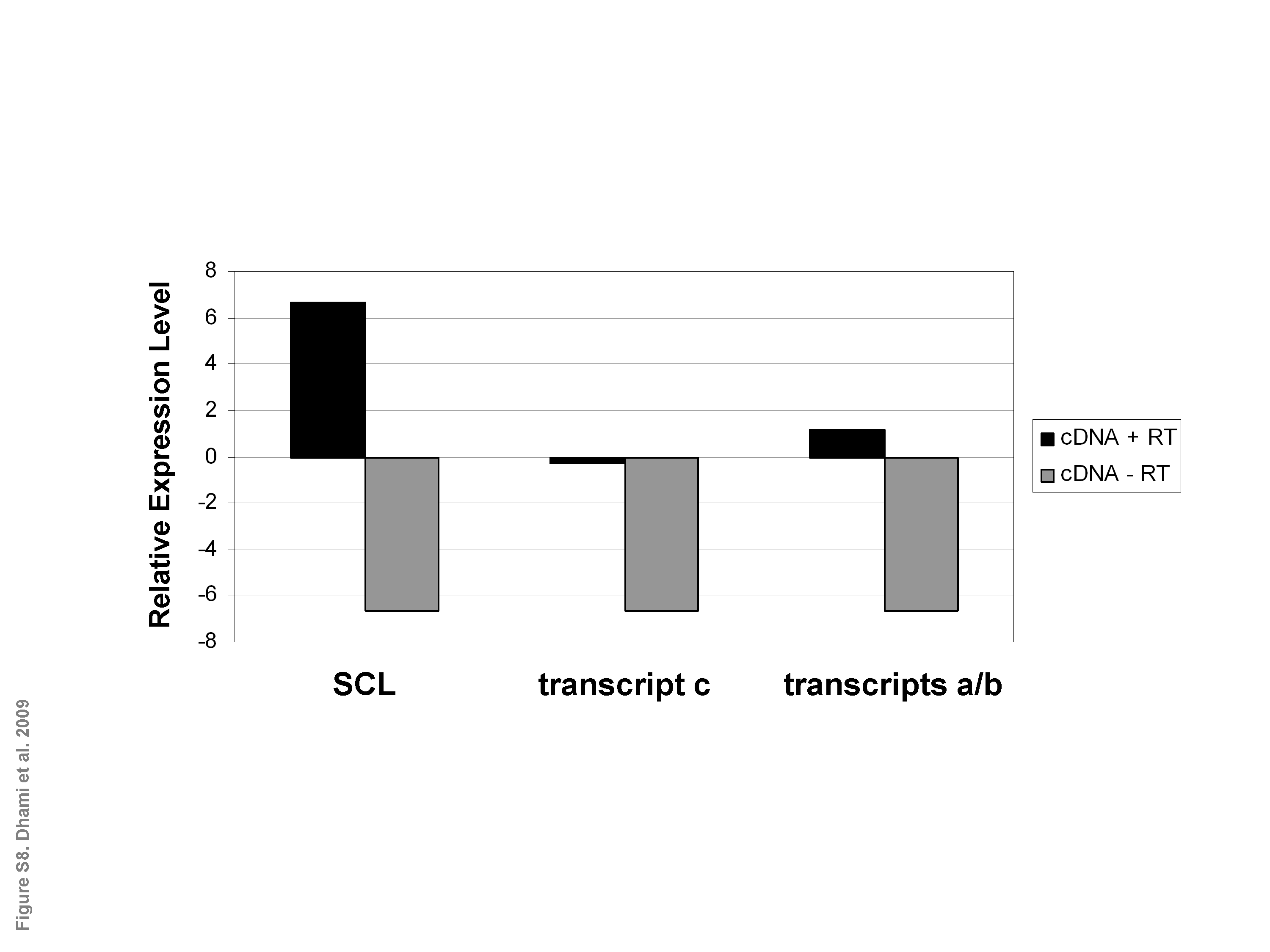

Supplement: Figure S8 — Expression of transcripts of unknown function at the +53 region in the K562 cell line. Histogram shows real-time SyBr green quantitative PCR results of transcripts a, b and c expressed in log10 scale relative to the level of expression of SCL (SCL is assigned an arbitrary level of expression). PCR amplifications from samples which were reverse transcribed into cDNA are shown as black bars. Amplicon for transcripts a and b was from coding sequence shared by both transcripts - thus expression results for these are presented collectively as a/b. Negative controls for PCR amplification in the absence of reverse transcription are shown as grey bars. (0.55 MB TIF) [file pone.0009059.s008.tif]

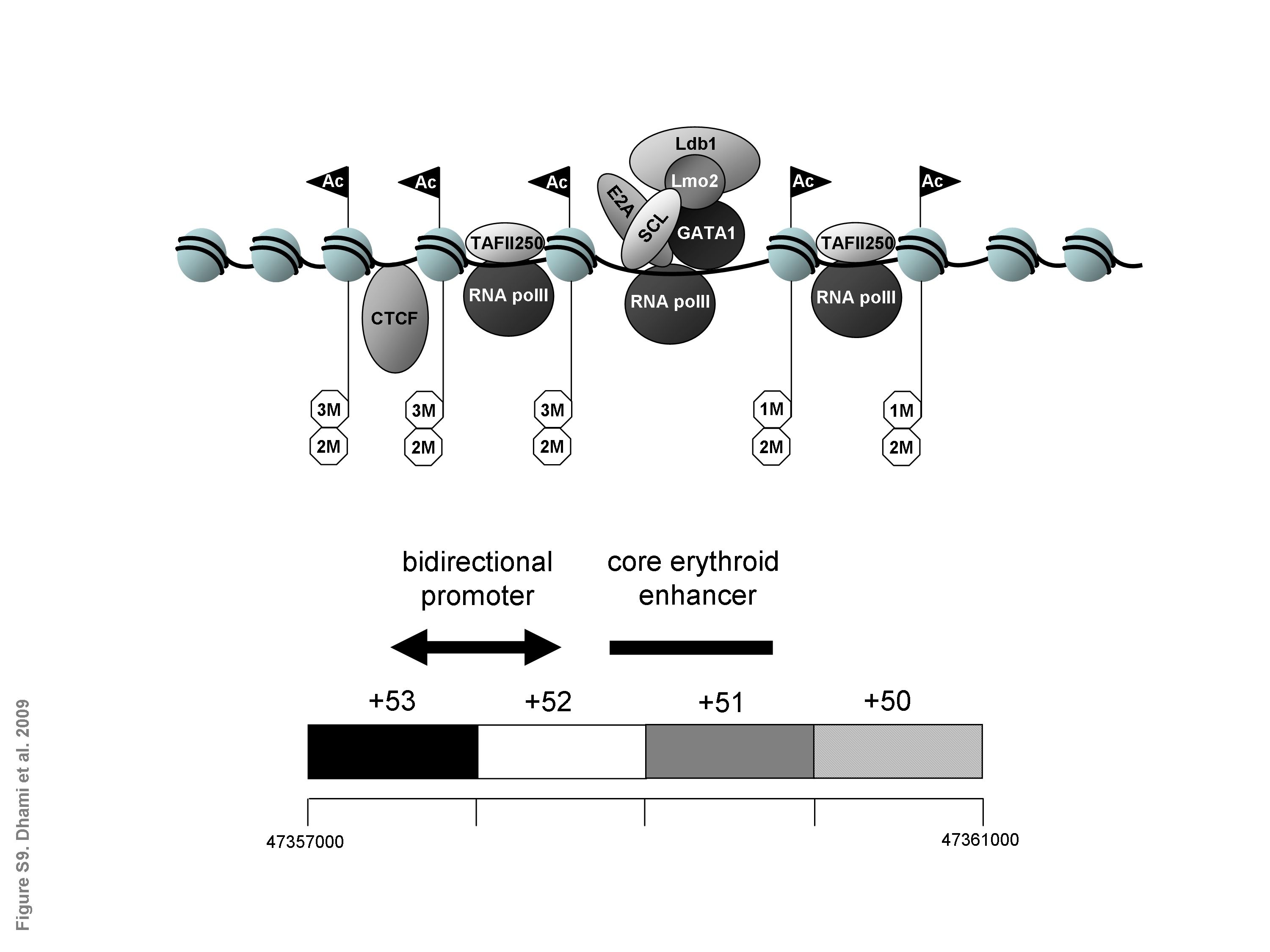

Supplement: Figure S9 — Schematic diagram of the biological events identified at the human SCL erythroid enhancer in the K562 cell line. At the top of the figure, DNA is shown as the black line wrapped around nucleosomes (blue/grey spheres). Regions of accessible chromatin are shown as regions with fewer nucleosomes per unit length of DNA. Location of transcription factors, RNApolII and TAFII 250 are shown by larger grey and black spheres and ovoids. Histone modifications are shown as flags on the nucleosomes and are histone H3 K9/K14ac (Ac), histone H3 K4me3 (3 M), histone H3 K4me2 (2 M), and histone H3 K4me1 (1M). The four kb block of genomic DNA (+50 to +53) shown at the bottom half of the figure is numbered in one kb intervals according to distance in kb from the SCL pro1a. Genomic sequence co-ordinates are from NCBI build 35. The core erythroid enhancer at +51 is shown as the black horizontal bar and the extent of the bar defines the region which drives enhancer activity in transient reporter assays (see Figure 4). The bidirectional promoter at +53 is shown as the black horizontal bar with arrowheads and the bar extent defines the region which has bidirectional promoter activity in transient reporter assays. (1.26 MB TIF) [file pone.0009059.s009.tif]
