# Supplementary material for: Genomic Approaches Uncover Increasing Complexities in the Regulatory Landscape at the Human SCL (TAL1) Locus
Source: PLoS One. 2010 Feb 5;5(2):e9059. doi: 10.1371/journal.pone.0009059 (PMC2816701; doi:10.1371/journal.pone.0009059)
Supplement: Table S1 — Array elements which show significant enrichments for each member of the SCL erythroid complex in K562 cells. The names of array elements shown in the third column are as described in Table S2. Significant enrichments derived as the mean ratio from multiple experiments are shown in log2 scale in the second column. Genomic sequence co-ordinates are from NCBI build 35. (0.09 MB DOC) [file pone.0009059.s010.doc]

| **Transcription Factor** | **Enrichment Ratio (Log2)** | **SCL Tiling Array Element** | **Chrom 1 Co-ordinate Start** | **Chrom 1 Co-ordinate Finish** |
| --- | --- | --- | --- | --- |
| E2A(E47) | 1.188 | HSTAL.80 | 47357585 | 47358097 |
|  | 1.354 | HSSCL/M46B | 47409142 | 47409449 |
|  | 1.558 | HSTAL.84 | 47359783 | 47360319 |
|  | 1.872 | HSSCL/M45A | 47410103 | 47410636 |
|  | 1.874 | HSSCL/M96A | 47359379 | 47359869 |
|  | 2.436 | HSSCL/M97A | 47358089 | 47358579 |
|  | 3.024 | HSTAL.82 | 47358481 | 47359020 |
|  | 3.537 | HSSCL/M96B | 47358901 | 47359347 |
| E2A(E12) | 1.698 | HSSCL/M48B | 47406584 | 47407045 |
|  | 1.705 | HSTAL.175 | 47419510 | 47419958 |
|  | 1.848 | HSSCL/M36A | 47419380 | 47419780 |
|  | 1.965 | HSSCL/M46B | 47409142 | 47409449 |
|  | 2.316 | HSTAL.84 | 47359783 | 47360319 |
|  | 3.007 | HSSCL/M97A | 47358089 | 47358579 |
|  | 3.118 | HSSCL/M45A | 47410103 | 47410636 |
|  | 3.16 | HSTAL.82 | 47358481 | 47359020 |
|  | 3.207 | HSSCL/M96A | 47359379 | 47359869 |
|  | 3.573 | HSSCL/M96B | 47358901 | 47359347 |
| GATA1 | 1.061 | HSTAL.164 | 47410476 | 47410942 |
|  | 1.155 | HSSCL/M36A | 47419380 | 47419780 |
|  | 1.177 | HSSCL/M97A | 47358089 | 47358579 |
|  | 1.223 | HSSCL/M46B | 47409142 | 47409449 |
|  | 1.288 | HSTAL.175 | 47419510 | 47419958 |
|  | 1.307 | HSSCL/M96A | 47359379 | 47359869 |
|  | 1.308 | HSSCL/M46A | 47409451 | 47409818 |
|  | 1.327 | HSSCL/M53A | 47402147 | 47402619 |
|  | 1.335 | HSSCL/M52B | 47402649 | 47403022 |
|  | 1.79 | HSSCL/M38B | 47416739 | 47417127 |
|  | 2.061 | HSTAL.170 | 47416739 | 47417312 |
|  | 2.481 | HSSCL/M45A | 47410103 | 47410636 |
|  | 2.711 | HSTAL.82 | 47358481 | 47359020 |
|  | 3.759 | HSSCL/M96B | 47358901 | 47359347 |
| LDB1 | 0.8071 | HSSCL/M15A5 | 47440134 | 47440817 |
|  | 0.8237 | HSSCL/M98A | 47357176 | 47357673 |
|  | 0.9294 | HSTAL.175 | 47419510 | 47419958 |
|  | 0.9421 | HSSCL/M36A | 47419380 | 47419780 |
|  | 0.9591 | HSSCL/M95A | 47360136 | 47360486 |
|  | 1.129 | HSSCL/M46B | 47409142 | 47409449 |
|  | 1.157 | HSTAL.165 | 47410918 | 47411429 |
|  | 1.527 | HSTAL.80 | 47357585 | 47358097 |
|  | 1.708 | HSSCL/M46A | 47409451 | 47409818 |
|  | 1.715 | HSTAL.84 | 47359783 | 47360319 |
|  | 1.788 | HSSCL/M97A | 47358089 | 47358579 |
|  | 1.797 | HSTAL.164 | 47410476 | 47410942 |
|  | 2.269 | HSSCL/M96A | 47359379 | 47359869 |
|  | 2.672 | HSSCL/M45A | 47410103 | 47410636 |
|  | 3.61 | HSTAL.82 | 47358481 | 47359020 |
|  | 4.822 | HSSCL/M96B | 47358901 | 47359347 |
| LMO2 | 1.889 | HSSCL/M96B | 47358901 | 47359347 |
| SCL/TAL1 | 1.151 | HSSCL/M52A | 47403471 | 47403807 |
|  | 1.167 | HSSCL/M45A | 47410103 | 47410636 |
|  | 1.277 | HSSCL/M46A | 47409451 | 47409818 |
|  | 1.603 | HSTAL.82 | 47358481 | 47359020 |
|  | 2.219 | HSSCL/M96B | 47358901 | 47359347 |
