# Supplementary material for: Genomic Approaches Uncover Increasing Complexities in the Regulatory Landscape at the Human SCL (TAL1) Locus
Source: PLoS One. 2010 Feb 5;5(2):e9059. doi: 10.1371/journal.pone.0009059 (PMC2816701; doi:10.1371/journal.pone.0009059)
Supplement: Table S2 — Oligonucleotide primer pairs used to PCR amplify array elements for the human SCL tiling array. Amplicon names used in our laboratory are shown in the first column. Amplicon sizes and genomic sequence co-ordinates are from NCBI build 35. (0.60 MB DOC) [file pone.0009059.s011.doc]

| **Amplicon Name** | **Primer 1 (5'→3')** | **Primer 2 (5'→3')** | **Amplicon Size (bp)** | **Chrom 1 Co-ordinate Start** | **Chrom 1 Co-ordinate Finish** |
| --- | --- | --- | --- | --- | --- |
| HSTAL.1 | CATGATATTCATCCCCTGCC | TTCTGAATGATGACCATGCC | 499 | 47262288 | 47262786 |
| HSTAL.2 | TTCAGCTCTCAAGGCCAAAT | TCCTAAGTCCCCAAAAACCC | 504 | 47262613 | 47263116 |
| HSTAL.3 | AATGACAAGTCAAGGGGGAA | AAAAATGGCCTAAGCCTGAA | 457 | 47267138 | 47267594 |
| HSTAL.4 | ATTTGAGCACCAGTCTCTACCA | CCTTCCGGTCCTGGATTACT | 459 | 47271823 | 47272281 |
| HSSCL/M183A | CCAGCGCCTTTTCTGAGTAGTAT | TACTGTCCCTTACCAACTCAGGAAC | 218 | 47272114 | 47272331 |
| HSTAL.6 | GTCTTTTTCCAAGCCCACAA | AGGTTGAAGATGCTTGGCAC | 507 | 47272825 | 47273331 |
| HSTAL.7 | GCCAAGCATCTTCAACCTTT | ATTTTGCAGTGCCCTGTTCT | 447 | 47273314 | 47273760 |
| HSSCL/M182A | TTTGCAGTGCCCTGTTCTTAGTA | ATCTCATGCCATTTCCGTTGTAC | 284 | 47273475 | 47273758 |
| HSTAL.8 | CTAAAACTGCCTGCAATCCC | CAACAGAATTCAACTGGGCA | 417 | 47276178 | 47276594 |
| HSTAL.9 | TCTGAAGCAGATCTCCAGGC | GGCAGACTATTCTTTCCGCA | 439 | 47276807 | 47277245 |
| HSSCL/M178A | GTGATAAGGAAGAGGGGTGTGAAT | AGCAGAGATGCCGAGATGAAAT | 488 | 47276919 | 47277406 |
| HSTAL.10 | GTCACAGGAGCCCTGGAATA | CCTCTTTCTCCCTCTCTGGG | 539 | 47283559 | 47284097 |
| HSTAL.11 | ATCACCTTTCCAGATGGACG | GCTACATCATGGCTCCAGGT | 511 | 47283920 | 47284430 |
| HSSCL/M171A | TGTTGCCTTTCAATCCATCTTG | TGAGGGGGCCTTCGTGAA | 405 | 47284183 | 47284587 |
| HSTAL.12 | CATTGGTGGAATGCAAGATG | GCAAAGATCCCCATGATAGC | 559 | 47284553 | 47285111 |
| HSTAL.13 | CACATAATATTCCGCCCACC | GCCTAAAACTGAGTCAGCCG | 452 | 47285213 | 47285664 |
| HSSCL/M170A | GAAAGCTCTGGAGGGCAAGAA | TCCCCCGACCCATACAAAA | 399 | 47285443 | 47285841 |
| HSTAL.14 | CTTTACATTCCTTTCCGCCA | AGCAAATGAAGAGAAGGGCA | 561 | 47285797 | 47286357 |
| HSTAL.15 | CCTTCTCTTCATTTGCTGGC | AAGTGGCAACAGAATCACCC | 467 | 47286341 | 47286807 |
| HSSCL/M168B | TGCAGCCACCCTCTCATTG | TCTCAGTTATTGCCTTTGTCAATCT | 383 | 47286641 | 47287023 |
| HSTAL.16 | ATTCTGTTGCCACTTGTCCC | TGGACATCCCCTTTTCTCTG | 470 | 47286793 | 47287262 |
| HSSCL/M168A3 | AAAAAAGCATGCACATTATCTAACAAA | AAGGGGATGTCCACTCTGCTG | 494 | 47287249 | 47287742 |
| HSSCL/M168A | TTATTTAGGGGATTTCATGGAACAC | ACAATGCAGATCCCTTTTCACTAA | 625 | 47287327 | 47287951 |
| HSTAL.18 | TGGCCTAGTATGTGCCATGA | CAGAACAGACTGCCCATTGA | 516 | 47287855 | 47288370 |
| HSSCL/M167A | AATGGCAACTGGAACACCATAAC | GAGACTCACCCAAAGGAGCTAATAA | 447 | 47288277 | 47288723 |
| HSTAL.20 | TTGCCACTAAAGCAGCATTC | TCCTTTTCAGGGGCTCTACA | 439 | 47289636 | 47290074 |
| HSSCL/M165B | TTTTCAGGGGCTCTACAATTTTTC | ATCAGTTGGGGAAAGAATAAAAGAG | 324 | 47289748 | 47290071 |
| HSTAL.21 | TGTAGAGCCCCTGAAAAGGA | CAGGGTGGAGATGATATGGG | 521 | 47290055 | 47290575 |
| HSTAL.22 | CAGAATCTTCGCTCATGCAC | GAAAGCAGAGAGCAAGGTGG | 400 | 47293173 | 47293572 |
| HSSCL/M161A | CCCAAAAGCCTACCACTGAAAA | TGGGAAGTAGAGGGGTGTGATT | 675 | 47293506 | 47294180 |
| HSTAL.24 | CGGGTTTACTGTGAAGGGAA | GTCAGCTAACTGAGGGCCTG | 495 | 47294020 | 47294514 |
| HSSCL/M160A | TAAGCCCCGATCAGAAAGTCC | CATGACAGGCCCAAAGACTACA | 426 | 47294670 | 47295095 |
| HSTAL.26 | CCACAGTTTGCAGGTCTTGA | AAGCGGAGCAGAGTTAATGC | 479 | 47295048 | 47295526 |
| HSSCL/M159B | AACTATGCTTGAAAGCCACTCTACC | GCAGCATTTTGCCATAATTGAG | 437 | 47295470 | 47295906 |
| HSTAL.28 | GCCACCTTCTGAGTCCCATA | CCCACCCTACAACCTCAAAA | 479 | 47296007 | 47296485 |
| HSSCL/M159A | AACCTCTGCGTCCTAACTTGTGA | CCCCATGCTGGTGTGTGCT | 509 | 47296301 | 47296809 |
| HSTAL.30 | TCCAGCTGTCACTCATGGAT | GCATGTGGGAAAGTGATAAAAG | 583 | 47305418 | 47306000 |
| HSSIL/M64A | CACAAGCACACTTTTCTAAGAGTTACA | GCAGCCCCTAAACCTTCCAG | 304 | 47305922 | 47306225 |
| HSTAL.31 | GAGCTGCCCTGGATAGTGAG | TCCCTACTTGGATCAGCACC | 561 | 47306091 | 47306651 |
| HSSIL/M65A | GGGAGGTTGGGTGGAATGAA | CTTGGGCCATTCAGAGACAGAG | 477 | 47306728 | 47307204 |
| HSSCL/M148A | GGGCCATTCAGAGACAGAGAAG | CCAGGATTTGAGGCAGAAAGAA | 410 | 47306792 | 47307201 |
| HSTAL.33 | GGATTAGGGAGCAGGAGGAA | GCCATAGAAAACTAAAATGGGG | 572 | 47306900 | 47307471 |
| HSSCL/M141A | CCAAGCCCTGAATCATTACTTTT | GCCAGGGAGGGTTAATTCATAG | 558 | 47314179 | 47314736 |
| HSTAL.35 | CCAGTCTCCCCAATGGAATA | GGGAGAACAGCTTCATCAGC | 448 | 47314549 | 47314996 |
| HSSCL/M140A | CTCCCCCACTGAGTTCCTCC | ATGGACAGGGGAATGTGGTAGA | 444 | 47314953 | 47315396 |
| HSTAL.37 | ATCCAGGAGGTAGGGAGGAA | ACTTAGCCAGTTGGTCCCCT | 569 | 47315364 | 47315932 |
| HSSCL/M139A | ACAGGAAGGGGATGACATGCT | GGAGGGCAATGCCTGATTG | 521 | 47315634 | 47316154 |
| HSTAL.38 | CCTCCAAGTGAGGAGACTGG | GATAGCAGTGCACAGGGGAT | 485 | 47316032 | 47316516 |
| HSTAL.39 | ATCCCCTGTGCACTGCTATC | AAAAGAGTCGGCCCTTCACT | 456 | 47316497 | 47316952 |
| HSTAL.40 | AGTGAAGGGCCGACTCTTTT | TCTAATGTCTGCCCACCTCC | 486 | 47316933 | 47317418 |
| HSTAL.41 | GGAGGTGGGCAGACATTAGA | CATATGGTGCTGGTGTTGGA | 500 | 47317399 | 47317898 |
| HSSCL/M137A | AGTCAGCCATTGTCTTCATGTTGT | GCAGGCAGCAGGGAGCTCA | 448 | 47317806 | 47318253 |
| HSSCL/M136B | ATCTGGGGCAATTTACGTTTC | CCTTCACTCCTGCTGCAAAGAC | 479 | 47318407 | 47318885 |
| HSTAL.45 | ACCAAGAACTGATGCTGCCT | TGACTGGTGACAATTTGGGA | 557 | 47319185 | 47319741 |
| HSSCL/M135B5 | GGGCAAATGCAGGAAAAGGA | TCAGACCCGAAATCCCATGG | 412 | 47319258 | 47319669 |
| HSSCL/M135B3 | CTGCACGGACCTAGGGATGA | ACAAATTCCTGGCTCCACGG | 476 | 47319283 | 47319758 |
| HSSCL/M135A5 | AAGGGCCTTGTGTTCACCATG | CTCCCTCCCAAATTGTCACCA | 438 | 47319716 | 47320153 |
| HSSCL/M135A3 | CTGTCCATGCTGCCACTGAAG | CCCAGCCTTCCACAATGACAT | 462 | 47319857 | 47320318 |
| HSTAL.47 | AGAGGGTTCCCCAGAGTTGT | AAGGGGCTAGTATGGGCACT | 468 | 47320067 | 47320534 |
| HSSCL/M134B | GGGGGCATGGACCTTCTTAA | TGCCCAGCACATTTTGATGA | 493 | 47320457 | 47320949 |
| HSTAL.49 | TCTCCTTGATGACCCTGGAC | ATGGCCTGGATGTAGGACTG | 465 | 47321016 | 47321480 |
| HSSCL/M134A | GGAGTCAGGGCAGGTACTTTCA | ACCGCCACACAAAGGAAGAG | 499 | 47321175 | 47321673 |
| HSSCL/M133A | CGTGGCCCTCAAACATGAA | AAGAGGAGCCTCCATGAACTGT | 470 | 47321845 | 47322314 |
| HSSCL/M132A | CCAGCCCTGCACATCTCAAG | GGCCTCCTGGGTGATGGA | 529 | 47322401 | 47322929 |
| HSTAL.53 | ATGGGGTCAGTGCAAAAGAG | CATGATACCTGTGGTGCAGG | 592 | 47322974 | 47323565 |
| HSSCL/M131B | TCAGAGGGTTGACCAGAGACTTC | TAGGTGTTTTGGGCCTTCTTTT | 448 | 47323461 | 47323908 |
| HSSCL/M131A | ACCGCAGCCATTCTGGAGT | TCAAATGGGCAGCCTGAATT | 665 | 47324064 | 47324728 |
| HSTAL.56 | TCTTGGAGAGAAAAGCCCAA | ATGTGTGTCTGGAGCACAGG | 598 | 47324602 | 47325199 |
| HSSCL/M130A | TCCAGCCCACATCCTAGCC | ATCATGTCGCTGCCACTAGAGAA | 515 | 47324950 | 47325464 |
| HSSCL/M129B | GACAGGGCCAGGGCTCTAAG | GATGCTGTCCCACCTGTGATG | 503 | 47325526 | 47326028 |
| HSSCL/M129A | GAGTGGGGGTCAGGAAGACAG | GCCAGGATGGGGTAGAAGTGT | 401 | 47326128 | 47326528 |
| HSTAL.60 | TCCCTGAATCTCCACCCAC | GATGACAATCAGGTAGGGGC | 591 | 47327280 | 47327870 |
| HSSCL/M127A | CTTAGCTCAGCCTTGCTGTGATT | TCAGCCAGACAATCCCTCTATCT | 407 | 47327924 | 47328330 |
| HSTAL.62 | GAGGGAGAAGCTCACACCTG | CTTTGAGGCTTTCCTGCTCA | 576 | 47328237 | 47328812 |
| HSSCL/M126B | GCCCAGGGTCAGCAGTACC | GAGGCTGCACTGAGCTGAGC | 409 | 47328778 | 47329186 |
| HSTAL.65 | GATCCCTCTCTCCTTGGGTT | GTTGCTGGATGTTTCCTGGT | 493 | 47338055 | 47338547 |
| HSSCL/M116A | GATGGCAGCCCCTAAACTTTC | CATCATCAACTGGCACAGTCCTC | 446 | 47338682 | 47339127 |
| HSTAL.67 | AGTCCCACAGTTTGCCATTC | TCAGCACCTGTATTTCACGC | 593 | 47338958 | 47339550 |
| HSSIL/M53A | GGGAGGGAAAGCAAAACTCAC | CAAGGGCACCAATTCAGAAACT | 467 | 47339446 | 47339912 |
| HSSCL/M115A | GCCACCCAGCAAAGAAGAAA | TGTCCCTACCCTTTGGCTTAGA | 439 | 47339676 | 47340114 |
| HSSIL/M53B | TTGGTGCCCTTGGCTATGAC | ATGTCACAGTTCAGTTGCTCAAGG | 305 | 47339901 | 47340205 |
| HSSCL/M113A | GCAGGACCATTTGCCTATTCTT | CTCATTTTCATGTTTGTAGTGGAATAGT | 298 | 47342174 | 47342471 |
| HSSIL/M55A | ATGATACAATGAAGATTGAAATGTGACA | TTCATCCTAACTGAAGGAGAATGATAAC | 325 | 47342204 | 47342528 |
| HSSIL/M57A | GTGGCCATGATGGGAAAATTA | TTCCCTTATCTATGCCATATCCATTA | 282 | 47343276 | 47343557 |
| HSSCL/M105A | TCGGGGTCACTGGGAAATG | CTAAAGAGGGTCAAGTTATCTTCTGG | 367 | 47350069 | 47350435 |
| HSSCL/M104A | TGATCGTCCCATGAATGTGAAGT | GCCATCAGAGCTTCCATTTTAAT | 471 | 47351208 | 47351678 |
| HSTAL.71 | AGTCCATAACACCAGGGCAG | AAACAGTGTGTGTGGAGGGG | 575 | 47351603 | 47352177 |
| HSSCL/M103A | GGGGGTAAGGGCACTTGTTT | ATACAGAGAACAGGAACTGGTATTACATAT | 391 | 47352054 | 47352444 |
| HSSCL/M102A | CGGGTCTCAGGATCTCCTTTT | ACAGCCAGCCAGGATGCTT | 418 | 47352494 | 47352911 |
| HSTAL.73 | GACAATGGCTAGTGGGCAAT | CAGCCATCGTGATTTTCCTT | 504 | 47352766 | 47353269 |
| HSTAL.74 | TCCTCTCGCAGATGTGAATG | TTTGTGAGGCAAAGACCACA | 508 | 47353496 | 47354003 |
| HSSCL/M101A | ACCACCTGCCCTCAGCTCAT | TGCAGGAGGGAGTGGAGCT | 271 | 47353827 | 47354097 |
| HSTAL.76 | GAAAACAGCACCACCACCAT | CAGAGTGAACTGAATTGGATGC | 509 | 47355636 | 47356144 |
| HSTAL.77 | ACATTCTGTACCTGCCAGCC | GAGTTCTGAAACAGCTGGGC | 525 | 47355951 | 47356475 |
| HSSCL/M98A | TGCCGATTGGTTCAAATTCC | CGGGACAGATGGCTGGAGT | 498 | 47357176 | 47357673 |
| HSTAL.80 | CAATTTTACCGGCAAAGGAA | GAGTTCCGTCTCCAGAGTGC | 513 | 47357585 | 47358097 |
| HSSCL/M97A | GTGCCCACCTCCTCTTTCTCT | ACGGAACTCCACAGCCTGTTT | 491 | 47358089 | 47358579 |
| HSTAL.82 | AAGGAAGCTTAGGAGGCAGG | CTGGAGAAGGCTGATAACGC | 540 | 47358481 | 47359020 |
| HSSCL/M96B | GCCCCCTTCTCATTCTGTCTT | CTCCAGCCCCGCCCTAGT | 447 | 47358901 | 47359347 |
| HSSCL/M96A | CGTGGCTGCCTGTGACTCA | TGCTGGGGAGCAAGGTGA | 491 | 47359379 | 47359869 |
| HSTAL.84 | GAACCTTGGTTGGGTCAAGA | TCACCTCTGTGACTGTTGGC | 537 | 47359783 | 47360319 |
| HSSCL/M95A | ACGGCAGAGCTGGAGGAGA | AAGCAAGGGGGCTGTGACA | 351 | 47360136 | 47360486 |
| HSTAL.85 | ACAGTCACAGAGGTGAGGGG | CCACTCTACCCTTGCTCCAG | 523 | 47360304 | 47360826 |
| HSSCL/M94B | GCCAGGGTGAGGGTAAGAGAG | GGGCTACTGGAGGGAGACAGA | 485 | 47360654 | 47361138 |
| HSSCL/M94A | AGCACCCCGATGTAACCTTCT | AGGGGCTTCCTGCATAATCC | 479 | 47361199 | 47361677 |
| HSSCL/M92A | TTCAGGGAGAGCCCACACTG | CCCCCTCGTCCAAGCAGA | 427 | 47362829 | 47363255 |
| HSTAL.92 | ACCAAGACTCCAAGAAGCGA | AGGTGGGGTCCTTGAAGAGT | 490 | 47364722 | 47365211 |
| HSSCL/M90B | CCTGCCTCCCCACAATGTC | CCCACCCTGCCTGCTCAG | 296 | 47364972 | 47365267 |
| HSTAL.93 | ACTCTTCAAGGACCCCACCT | TGTCCTGGTCCACTCCTCTC | 515 | 47365192 | 47365706 |
| HSSCL/M90A | TGGGGCCTCAACCTAATCTCT | GGCAGCCCAGAGCCTCTTT | 427 | 47365419 | 47365845 |
| HSTAL.94 | GAGAGGAGTGGACCAGGACA | AGAGGAAAGTCCTTTGGGGA | 409 | 47365687 | 47366095 |
| HSTAL.95 | AAGGACTTTCCTCTTCCCCA | ACCTGAGGGAGCAGTCTGAA | 470 | 47366082 | 47366551 |
| HSSCL/M88B | CAAGGTCTGGCTTTCATTCTACAC | AAGCAGTATTTGGGAGTCATTGTTT | 467 | 47366498 | 47366964 |
| HSTAL.96 | CCCTGTTTCCTCCTAGACCC | GAGAACAGTGCCCAGTGTGA | 578 | 47366602 | 47367179 |
| HSSCL/M88A | AGACTGAGCCCAGGTTGATTTC | TCACACTGGGCACTGTTCTCTATC | 569 | 47367160 | 47367728 |
| HSTAL.98 | AGTCTATTGGTGTCCGCCTG | AGGCACTGAAGCCGTAGTGT | 574 | 47367688 | 47368261 |
| HSSCL/M87A | TTCAGGCCCTCTATTGAGATCTTT | GCTGGCATTCGAGGTCATCT | 463 | 47367872 | 47368334 |
| HSTAL.99 | ACACTACGGCTTCAGTGCCT | AGGAACTCTGGCCATTCTCA | 536 | 47368242 | 47368777 |
| HSSCL/M86A | CCTGGGGACCTTCCATCTCT | TCTTGGCCCCAGATGTGGT | 505 | 47368414 | 47368918 |
| HSTAL.100 | GAGAATGGCCAGAGTTCCTG | GCATCACTCAACTCCAAGCA | 485 | 47368759 | 47369243 |
| HSSCL/M85A | TGTGGGGCTGCTGAGACCT | CCTCCAACCAGTGCCCTCA | 411 | 47370130 | 47370540 |
| HSSCL/M84A | GCTCTTTGCCTGGATTCCTCT | AACCATCAGGCCCCTTAGACA | 457 | 47370620 | 47371076 |
| HSTAL.103 | GCAGGCAAAGTTAGCCAGAG | TTTAAGGGCTGATGAATGCC | 555 | 47371010 | 47371564 |
| HSTAL.104 | GCAAACTTCTTTGCATGGGT | GTCCTTGCCCCACAGATAGA | 581 | 47371572 | 47372152 |
| HSTAL.106 | GATCAATCAACCCATCCTGC | TTTCCAGTCCCATAGTAGCCA | 471 | 47374482 | 47374952 |
| HSSCL/M78A | GGCACCTCCTTGATTGTACTCTTT | TGGGTGCAGCCGTGGAG | 520 | 47376719 | 47377238 |
| HSTAL.108 | GCTGCATAGACGCAGAAGTG | TGCTCACTGCACCCACTAAC | 593 | 47377186 | 47377778 |
| HSTAL.109 | GGAGCTCACAGGTTAGTGGG | GACTCCACATGCCCTGAAAT | 403 | 47377748 | 47378150 |
| HSTAL.110 | ATTTCAGGGCATGTGGAGTC | GCTTCTTGACTGAGCAAGGG | 445 | 47378131 | 47378575 |
| HSSCL/M76A | CTTCTCTCGCCACACTGTGCT | CCCTCGGACCCTTTCACACT | 559 | 47378586 | 47379144 |
| HSTAL.112 | GTAATTGGAGGCTTCCCCTC | GACTCCTTTCCTGGCATTCA | 440 | 47379037 | 47379476 |
| HSTAL.114 | TACAAGTCACATCCCTCCCC | TCCATCCTCTTCTCCCTGAA | 485 | 47385593 | 47386077 |
| HSSCL/M69A | GGCTGGCAGGCTGTGTGA | GGGCAGGATGGGACCATTAG | 634 | 47385726 | 47386359 |
| HSSCL/M68B | AGTCCCCAGCCAATCTTTCC | GCCCCAACCCAGCAGATG | 461 | 47386420 | 47386880 |
| HSTAL.116 | GGTGCTTAGGGTGATGGAAA | TATGCCTGGTGACAGCTCAG | 586 | 47386620 | 47387205 |
| HSTAL.117 | TGAGCTGTCACCAGGCATAG | AGAATCCAGGGTAGAGGGGA | 512 | 47387187 | 47387698 |
| HSSCL/M67A | CACAGCGAGGCTGCTTAGAGA | AGGGAGGGAACAGCAGGACA | 482 | 47387699 | 47388180 |
| HSTAL.119 | ATAGCACCGCAGTCTTGCTT | TGGAGTGCTGCCCTAGAAGT | 527 | 47388080 | 47388606 |
| HSSCL/M66A | CTGCCTGGGTCTCCCTCTG | CACCAGCAGATCTAGGTTCTAGCTT | 414 | 47389245 | 47389658 |
| HSTAL.121 | CTTCGAACGGATCACATCCT | ATACAGAGCCCTTCCACCCT | 421 | 47389762 | 47390182 |
| HSSCL/M65A | TGCTTGGGAATGGCAGAAGA | GCAAGTGGGGAGAGGTAGGAA | 509 | 47389839 | 47390347 |
| HSTAL.122 | AGGGTGGAAGGGCTCTGTAT | GTCAGGGCAGTCAATTTGGT | 500 | 47390163 | 47390662 |
| HSTAL.123 | ATTGACTGCCCTGACTGTCC | ATTGGGAGGCTGTTGATTTG | 575 | 47390648 | 47391222 |
| HSSCL/M64A | GGTGGGAGGCAGAATCATTGT | GAGGGGGAACACTGGCTTCT | 334 | 47391105 | 47391438 |
| HSSCL/M63A | GTCCTCCAGGGAAAAGAGCTG | CTCCCACCCATCTCCACTCTT | 507 | 47391431 | 47391937 |
| HSTAL.126 | ACCTCATCGTTTCTGCCTTG | ATGGTGAGATTTAGCCGTGG | 491 | 47392128 | 47392618 |
| HSSCL/M62B | GCCAAGAGGTGAGTTCAAGGAC | CCCTCAAGCCCTTAACCACAG | 369 | 47392495 | 47392863 |
| HSTAL.127 | CCACGGCTAAATCTCACCAT | GGCCATCAAGGTGAAAAAGA | 584 | 47392599 | 47393182 |
| HSSCL/M62A | TTATGCCTGGCTCTTAAGGATTG | GGTGCCAGTTCCACATGCTG | 479 | 47393085 | 47393563 |
| HSTAL.128 | GTGCCTGCATGACCTACTGA | TCAGCAGACATCCCCTCTTT | 404 | 47393307 | 47393710 |
| HSSCL/M61B | ATGAATTGCGTTGGTCTCTGC | GGAAGCTCACCCCAACACAA | 503 | 47393673 | 47394175 |
| HSSCL/M61A | CCAAAACAAGCCTGGTAGGAAA | GCAGGGGGCAAGTCTTCAG | 443 | 47394243 | 47394685 |
| HSTAL.131 | CCTGGCCTTGGAACAAGATA | AATCTCAATCCTGCAGTGGG | 503 | 47394753 | 47395255 |
| HSSCL/M60A | AGCTTGGGGCAACATTGTTC | CAGGCCAAAGCGGTTTACAA | 525 | 47395143 | 47395667 |
| HSTAL.133 | AGGACAAGCCACTAGGCTGA | TGCAGCTTTTGGTCATCAAG | 463 | 47395756 | 47396218 |
| HSSCL/M58B | TTCGTGAGCCCCATCTTCAC | CGTACGCCCATCAGCACAC | 415 | 47396529 | 47396943 |
| HSSCL/M58A | GTGCTTTCCCCCAACTCCA | CTCCAGGCCAAAAGCAGTGA | 461 | 47397098 | 47397558 |
| HSTAL.137 | TCCTCCTCCTGGTCATTGAG | GCCTCTTGGCACAAGTGAAT | 547 | 47397662 | 47398208 |
| HSTAL.138 | GCCAAGAGGCCTTACAGCTA | GGCTGTTTCTTCACAGCCTC | 462 | 47398199 | 47398660 |
| HSSCL/M56A | TCGATGTTAGGGAGAGAAGCAGT | TCCACAGGGCCTTGCAGTAA | 475 | 47398789 | 47399263 |
| HSTAL.140 | CTTGAACTTCTGGGCTGAGG | CCTGCAGTTGTGCTGTGTTT | 512 | 47399219 | 47399730 |
| HSTAL.141 | GACAGAGAGCATCCCTCAGC | CAGACATGTAGGCGAAGGGT | 549 | 47399791 | 47400339 |
| HSSCL/M55A | GGGGCTGGGATCTGCACA | ACAGGAATCCACATCAAGTCCTAA | 549 | 47400200 | 47400748 |
| HSSCL/M54A | CGATCCCCCATTCTAGGCAC | CCTTGGGCAGCCTTCCTAC | 475 | 47400853 | 47401327 |
| HSSCL/M53B | GGTGGGGGGCTCAAGACA | GGGCTAAGCGCCATTATGTG | 447 | 47401442 | 47401888 |
| HSTAL.145 | GGGGAAGGTCTCCTCTTCAC | GTGGTGACCAAGATGCACAC | 538 | 47401719 | 47402256 |
| HSSCL/M53A | CCGCCTGAGGACTGACCTG | CTCCCGCCCCAGAAACAA | 473 | 47402147 | 47402619 |
| HSSCL/M52B | GGAGCGCAGGGAGAGGAG | CCTCTGCCACCACCCTCAG | 374 | 47402649 | 47403022 |
| HSSCL/M52A | CAGGCACCCCTCCCTTCTC | ACGCCGTTCAGCAGGACC | 337 | 47403471 | 47403807 |
| HSTAL.150 | AGGGAAGAGGAGGGAACAAA | TCAGAGTCCAGCTGAGCAGA | 446 | 47403976 | 47404421 |
| HSSCL/M51A | GGGGTCACAAGGTGGTTTCAC | CGCCCAGCGGATCTTTACT | 505 | 47404306 | 47404810 |
| HSTAL.152 | TCACTTTGCTCAACCCTCCT | CCTGGGTGTCTAGTGGCAAT | 493 | 47404756 | 47405248 |
| HSSCL/M50A | TGGCATTCCCAAAGAAGGTGT | GTGCTGGGGCTCTTCCATC | 443 | 47404857 | 47405299 |
| HSSCL/M50A5 | TCCCAAAGAAGGTGTACAGCAGTC | CTGGGGCTCTTCCATCACCT | 434 | 47404859 | 47405292 |
| HSSCL/M50A3 | TTCCAGTGGCCTTGGTGTCTT | AAGGGCCAGAGTTTGTGAGTTG | 529 | 47404939 | 47405467 |
| HSTAL.153 | CCAGAAGGGAGAATGGATCA | GCAGTGAGGTAGGGAACCAA | 407 | 47405181 | 47405587 |
| HSSCL/M49B | TGGGGCATGGACCTCTGG | CAAGGCCACTGGAAATGCAC | 505 | 47405455 | 47405959 |
| HSSCL/M49A | GGTGGGGAGGCAGGAACA | AAGGAACGCCCGAGAGATTT | 447 | 47406027 | 47406473 |
| HSSCL/M48B | AGATCGCCCAGGACCACAC | ACAGCCGCCCCAAAGTTAC | 462 | 47406584 | 47407045 |
| HSTAL.156 | CTGTCCTGAGCCTTCCTCAC | GAGGGGTTGTTGTTGCTGTT | 515 | 47406802 | 47407316 |
| HSSCL/M48A | CCCCTTCCCAACTCCATTTC | CCCCGACCAACCAGTCCA | 497 | 47407155 | 47407651 |
| HSTAL.157 | AACAGCAACAACAACCCCTC | ACCCCGAAAAATCGAAAATC | 557 | 47407297 | 47407853 |
| HSSCL/M47B | CCGCTACAGCCGTTTTCTAAG | TTCGGGGTTTAGACAGAGTAAGG | 497 | 47407846 | 47408342 |
| HSSCL/M47A | CGCCTGTGCCTAGGTGTTTG | GCTGCGCTCTGGGATGATTA | 491 | 47408395 | 47408885 |
| HSSCL/M46B | GCGGTTGGGGTGTGTAGATG | CCACAGAAGGGCAGCAAACA | 308 | 47409142 | 47409449 |
| HSSCL/M46A | GGCCTCCCACCGAATTCTT | CAGGCACACCACACTCGGA | 368 | 47409451 | 47409818 |
| HSSCL/M45A | CCATGTGGGGAGCAGTGTTC | GCCATTATGGGCCAAATGATT | 534 | 47410103 | 47410636 |
| HSTAL.164 | CCCTTCCCTACCTCTTCACC | AAAGACCCTGGTCTCTGCAA | 467 | 47410476 | 47410942 |
| HSTAL.165 | ACTCATTGCAGAGACCAGGG | CCAGTTCTCAGGCTCCAAAA | 512 | 47410918 | 47411429 |
| HSSCL/M44A | CCTTATGGTAGGGGGGAACTAGA | TGCCAGGCTGATTTGTCCTT | 316 | 47411366 | 47411681 |
| HSTAL.166 | GGCTTCTCCTTGGTCATCAG | CCCACCTTCAAACCAAACAA | 599 | 47411507 | 47412105 |
| HSTAL.167 | CATTAAATGTCCCCAGCCAG | TGTTCTTGTCCCCTGCTCTT | 569 | 47412831 | 47413399 |
| HSSCL/M42A | TCGGCTGCTCATCAGAGTGC | GGCGCTGCCTGAAATAGCA | 444 | 47413153 | 47413596 |
| HSTAL.168 | ATGCATGCACTCTGATGAGC | TGGAAGCACAGACGTGACTC | 595 | 47413571 | 47414165 |
| HSSCL/M40A | AAACCGGGGGATATCACAATG | GCGGAAGACCAAAGCATACAG | 556 | 47415098 | 47415653 |
| HSSCL/M38B | GAATGCACATGCGCTTAAAATAG | TGGCCCCACATCAATCTTATG | 389 | 47416739 | 47417127 |
| HSTAL.170 | TGGCCCCACATCAATCTTAT | GGGAATGTGGCAAGAGAATC | 574 | 47416739 | 47417312 |
| HSSCL/M38A | AGGAGAGGGCCACAGCTTTT | CCCCATAGAAGCAAGGCATG | 259 | 47417271 | 47417529 |
| HSTAL.171 | TCACCGTTCAGGAGACACAC | AAGGGATTCTCCTGCCTCTC | 568 | 47417423 | 47417990 |
| HSTAL.172 | GAGAGGCAGGAGAATCCCTT | CCTCTAGCAGGCACACACCT | 533 | 47417971 | 47418503 |
| HSTAL.173 | AAGAATCCATTTGCACTGCC | AGTCCCAGGGCAACCTAACT | 588 | 47418453 | 47419040 |
| HSSCL/M36B | TTTCCCTGTGGCTGGTTTCTA | GGGGGAAGGGTGAACACCA | 425 | 47418970 | 47419394 |
| HSSCL/M36A | TGGGAGCAGCAGGTTGAAAT | CCAGCCACAGGGAAATGTGA | 401 | 47419380 | 47419780 |
| HSTAL.175 | GGTTTGGGGATGGGTAGACT | CCCATGCTCTTAACCTGAACA | 449 | 47419510 | 47419958 |
| HSTAL.176 | TGTTCAGGTTAAGAGCATGGG | CATTGGGGATCAGTAGGGTTT | 451 | 47419938 | 47420388 |
| HSSCL/M35A | TCGGACTCATATGTGCAGAATCAT | CCAATGCGGCAAGCAAATA | 320 | 47420383 | 47420702 |
| HSTAL.178 | ACCAGAGAAAGCGCTATGGA | AATCAAACCCAGGCCTCTCT | 536 | 47420920 | 47421455 |
| HSTAL.179 | TGGAGGCAAAGGGAGTAATG | TCCTGGTCAGAGGCTGAACT | 468 | 47421331 | 47421798 |
| HSSCL/M33A | GAGTTGGGCTTTGAAAGATTTTG | CCCCAGACCTCCCATGAAGA | 421 | 47421718 | 47422138 |
| HSTAL.181 | CCCAACTCCAGTGCTCTCTC | ACAGAGGCGGTGACATTTTC | 422 | 47422131 | 47422552 |
| HSTAL.182 | GAAAATGTCACCGCCTCTGT | AAGGCAGAACCTTCCCTAGC | 577 | 47422533 | 47423109 |
| HSSCL/M32A | TGCCCTTTAGCCAAGACCAG | AGTGGCTGCATTGGGCTAGA | 510 | 47422804 | 47423313 |
| HSSCL/M31A | TCCTCCAGTCCTGCAGCATAC | TCATTCATGTTGGCTTTTCATCA | 394 | 47424426 | 47424819 |
| HSTAL.184 | ACCTTCCAGAGTAGGCTTCG | CACTGGGAGCCCTGTAATGT | 555 | 47425549 | 47426103 |
| HSSCL/M29A | TAAGGCTTTTCTGATGAAGTTAAATTG | ATTACAGGGCTCCCAGTGATAAA | 521 | 47426086 | 47426606 |
| HSSCL/M27A | CCAGCATGTTGCATCTCTGCA | GACAACAAAGGAAAATAGTCAATCTAGAC | 387 | 47427821 | 47428207 |
| HSTAL.186 | TACACCAAAACCAACCCACC | TCCAGCTCTAAGTCTCAACTGC | 587 | 47427971 | 47428557 |
| HSSCL/M26C | TAGGCACCTTCTTAGATGTAAAACG | GCAGTTGAGACTTAGAGCTGGATAGTA | 345 | 47428536 | 47428880 |
| HSTAL.187 | AATGATCAGGAGCCTTGTGG | GCTTGGTGGTCAGAAAGAGC | 469 | 47428735 | 47429203 |
| HSSCL/M26B | CACAGTGGGGCTTAGTTTAATTTC | TCATCTGCTTTAGCGTTTCAGAAG | 488 | 47428896 | 47429383 |
| HSSCL/M26A | AGGACCATGAACTTAAAGCTGAAGA | GGAGAAGGCTGAATGGGTCAC | 328 | 47429403 | 47429730 |
| HSTAL.189 | TTCAGCTTTAAGTTCATGGTCC | TTGGGGCCTTGTATTTGTTC | 519 | 47429708 | 47430226 |
| HSSCL/M25A | CCATATCCAGAGACTGGTTGATGTT | AATAATAGCCTAGAAGGTAATCCATGAT | 349 | 47430116 | 47430464 |
| HSTAL.190 | GGCCCCAAATGTAAAAGTCA | TGCCAACATTAGCAAATGACA | 485 | 47430219 | 47430703 |
| HSTAL.191 | ATGGCTTTCTACATCATCCCA | TGGCATCACTGGTTTCAGAG | 505 | 47431421 | 47431925 |
| HSTAL.192 | AAGAAAGCAGGTCTAATTTGGG | TGGCTAGCTTGTGTTAGGCAT | 436 | 47432200 | 47432635 |
| HSTAL.193 | GCCTAACACAAGCTAGCCAA | CCCATAAATTGTTGACATGTGG | 414 | 47432617 | 47433030 |
| HSTAL.194 | TTAGATGTGCAGCCACAAGG | ATGGAGATTGTGGGTTCGAG | 512 | 47432956 | 47433467 |
| HSSCL/M22A | TCCGCCAGAAGAGTGGCTAA | GTTTTTCAACTACTAAGCAGATAGCCA | 288 | 47433323 | 47433610 |
| HSSCL/M22A3 | AGAATGCCGCAGAGATTAAGAGA | AGCCAGGAAGCAAGGTAAAGAAG | 206 | 47433344 | 47433549 |
| HSTAL.195 | CTCGAACCCACAATCTCCAT | AGGTCATCACCGACGCTTAC | 490 | 47433448 | 47433937 |
| HSSCL/M21A5 | CCGACGCTTACAGTGAAGGTTC | TCTGCGGCATTCTATTAACAGGA | 391 | 47433537 | 47433927 |
| HSSCL/M21A3 | CTTAGTGGGCCTTCAATGAGTCA | TCCCCTGCCTTAGCCACTCT | 409 | 47433581 | 47433989 |
| HSSCL/M21A | AATCCTCATTGCCTCTACCACTAAC | CTTCTGGCGGAATGATGTGC | 415 | 47433600 | 47434014 |
| HSTAL.197 | AGGGGCCATTTTGATGTAGA | TCCTAACCCTTCCCCTCTTG | 568 | 47435305 | 47435872 |
| HSSCL/M17A | GCCCATCATTGGCTAGATGAA | CAGAAAATATGCCAGCCTTAATAATAT | 492 | 47437422 | 47437913 |
| HSSCL/M17A5 | TCAGGGCCTACCATATGCATG | CATGACCAGTTGGGCACATTC | 290 | 47437526 | 47437815 |
| HSSCL/M17A3 | ATTGTGGCAATCTCATCAGAAAAGT | TTAGATGGCACGACTGTGTAACTGTA | 305 | 47437573 | 47437877 |
| HSTAL.199 | TATCCAGCCAGGAGTCATCC | CTCCAAGGAAACTGAGCAGC | 496 | 47437707 | 47438202 |
| HSTAL.200 | CTGCTCAGTTTCCTTGGAGG | TTCCCTGGCCTTGTTGTATC | 447 | 47438184 | 47438630 |
| HSSCL/M15C | TTGTCCATTTGCGAGAAGGC | GTAATTCTCCAAGTATTTTCGAGGAC | 311 | 47439625 | 47439935 |
| HSSCL/M15B5 | AAATGTGATCTGGGACTTAAAGGATAG | AATTAGGCCTTTATGATGTTCACAGAG | 286 | 47439721 | 47440006 |
| HSTAL.201 | AGGCCTTTATGATGTTCACAGA | TGAGAGCCTAGGGGAAACAA | 421 | 47439726 | 47440146 |
| HSSCL/M15B3 | AAGGGGTAATTCTGGTTGGAGG | TTTCTGCATATTTCATTCCCTGAG | 249 | 47439800 | 47440048 |
| HSSCL/M15B | TTGAAAATTGGAAAGTAGTGGGATAT | GCCTTCTCGCAAATGGACAA | 380 | 47439916 | 47440295 |
| HSSCL/M15A5 | TCCTTCCCAGCAGCTCTTCTCT | CCTAGGCTCTCAAGGGCACAG | 684 | 47440134 | 47440817 |
| HSTAL.202 | CTCAAGGGCACAGAGTGTCA | AATGCAACCCTTGCTTCATC | 510 | 47440143 | 47440652 |
| HSSCL/M15A | ACAACCAGGTGCTGCTTGAGTC | TTTGGTTTGAATTAGGCATAATATCA | 581 | 47440333 | 47440913 |
| HSSCL/M15A3 | TGGGGTGTTTATGTAGCATCATC | ACCTTCTCAAAAAGATGAAATGCT | 396 | 47440556 | 47440951 |
| HSTAL.203 | AGAGAAGAGCTGCTGGGAAG | AGCTTGTTTTCCAGGCTTCA | 559 | 47440797 | 47441355 |
| HSTAL.204 | GAAGCCTGGAAAACAAGCTG | TGCTTCCTAGGTTAGACACCAA | 453 | 47441337 | 47441789 |
| HSTAL.205 | TGGTGTCTAACCTAGGAAGCAA | ATTTGCCTTGCAGCTTCATT | 512 | 47441769 | 47442280 |
| HSSCL/M13A | CTCTGTCGATCCCAACACATAACTA | AGGTATGCCCAAAGCACAGC | 404 | 47442151 | 47442554 |
| HSTAL.206 | GATCCCAGAGAAGCACCAAA | AATGGGGGTTTTCAGATTGG | 571 | 47442428 | 47442998 |
| HSSCL/M11A | GGGGCAGGCAGTGGATTACT | ATATGGAAATTACTATGATCCTTACCCT | 263 | 47443770 | 47444032 |
| HSTAL.207 | TCCTTACCCTGCAGGAACTC | TCCCAGGATAATAGCACATGAA | 538 | 47443788 | 47444325 |
| HSSCL/M8A | TTAACAAAGGTTTGGCAGAGTATTATT | GGATACAGAAAGTGGCTGGTTAAAT | 305 | 47447329 | 47447633 |
| HSSIL/M70A | AAAGTGGCTGGTTAAATTCTTCTTC | AGGTTTGGCAGAGTATTATTTTTAAAG | 290 | 47447337 | 47447626 |
| HSSCL/M7A | CCTGCCTGATATTACAGATATTACAGATG | TCAGAAATGCCAGGAAGAGGTAG | 313 | 47448021 | 47448333 |
| HSSIL/M71A | GCCAGGAAGAGGTAGGTGGAA | ACCTGCCTGATATTACAGATATTACAGA | 306 | 47448029 | 47448334 |
| HSSCL/M5B | CTTCCCCTGGCTTGCACAT | CCAGAATTGGGTCCAGCCTAG | 289 | 47449530 | 47449818 |
| HSSIL/M72A | AATTGGGTCCAGCCTAGAAAAGT | GGCGCTTTGTAATTCATTATTAAAC | 485 | 47449534 | 47450018 |
| HSSCL/M5A | AGTGATAATGGCTTTCTATATTGAATAAGT | CAAGCCAGGGGAAGACTGTG | 307 | 47449805 | 47450111 |
| HSTAL.213 | GACCACGGCCTGTGATCTAT | CGCTTTGGATAGGAACACCT | 452 | 47456154 | 47456605 |
| HSTAL.214 | AGGTGTTCCTATCCAAAGCG | CTGACGTCCTTCCTCCACAT | 557 | 47456586 | 47457142 |
| HSTAL.215 | ATTGGTGCTGGGGAGAAAG | TTTTTCAGAACCTCTGTTGCAG | 400 | 47457079 | 47457478 |
| HSSIL_GAP/M39A | TTTTTTGCCTCTAATGAAAAGTCTG | GGTTCTGAAAAAAGGGAACATTT | 203 | 47457467 | 47457669 |
| HSTAL.217 | AGCAAACCTGTGCCTGAAGT | CCCCACTGCCATCTTACTGT | 450 | 47457926 | 47458375 |
| HSSIL_GAP/M38B | ACCCTCCACACTTAATTCTAGGC | GGAGATAGTCCCATCATTCCATTAT | 498 | 47457991 | 47458488 |
| HSSIL/M81A | TGCAACCCCAGCTTTATGATG | CTGCCCATATCTCCCTGAGGT | 625 | 47458132 | 47458756 |
| HSSIL_GAP/M38A | TGTTGGATGGCAATTTCATAGAATC | ATATCATGAGATGGCCCATTATGAG | 313 | 47458549 | 47458861 |
| HSSIL/M80A | CCAACCTGTGGGTCATTTTAGAA | CTCTGCAAAAAATAAAATTGTCACAA | 299 | 47459241 | 47459539 |
| HSSIL_GAP/M37A | TCACTAGAGCTTGACATTGGTTTTT | AAATTGTCACAAGCAGAATGTTTTC | 390 | 47459255 | 47459644 |
| HSSIL_GAP/M36B | AAAGAAAACACTGGATTACCAAAGG | GGTCTCTAACCACAAAAGTGAATGC | 213 | 47459993 | 47460205 |
| HSSIL_GAP/M36A | GCATGCCATCTGTGATACAATG | GGACCAGCCAGCCTTAATAAAC | 344 | 47460327 | 47460670 |
| HSSIL_GAP/M35B | GGGTGTTGGAGAAGAGCTTTGT | TGCATCAGGCTTTTTTACATCCT | 362 | 47460757 | 47461118 |
| HSSIL_GAP/M35A | GTCAACGGTTTTGTGTGTATATTCATC | AACACCCAAGGCAGCTTGAG | 444 | 47461112 | 47461555 |
| HSSIL/M78A | AATTCCATGCCACAACTCAGGT | CACCCAAGGCAGCTTGAGATAC | 395 | 47461114 | 47461508 |
| HSTAL.223 | TACCTGAGTTGTGGCATGGA | TGGCCTAAGCTTTAATGAGGA | 540 | 47461486 | 47462025 |
| HSTAL.224 | TGCTGACCCAAGAAGTCACA | TTCCTCTCCTCAAGCTGCAT | 579 | 47463484 | 47464062 |
| HSTAL.225 | ATGCAGCTTGAGGAGAGGAA | GGTCCTGAAATTGATCACCC | 462 | 47464043 | 47464504 |
| HSSIL_GAP/M31A | GGACAGTTTACAAAAGAGGGTTACAGA | TTCTGCATTTTGGCTTTCAGC | 208 | 47465287 | 47465494 |
| HSTAL.229 | CTGAGTGGACACGTGGTTATTT | TGTGTGGGAATAAGAAATGGTT | 598 | 47465838 | 47466435 |
| HSTAL.230 | TCCCACAAAGTTAACCTTCACA | AGATTGGGAGACTCACATGAAG | 556 | 47466805 | 47467360 |
| HSSIL/M51B | TTTATGAATGCTTCCCTTGTGATG | CCCACAAAGTTAACCTTCACAAGT | 407 | 47466806 | 47467212 |
| HSTAL.231 | CTTCATGTGAGTCTCCCAATCT | AGATTCACCCACTCCAGGTG | 577 | 47467339 | 47467915 |
| HSSIL_GAP/M28A | CATCTTTTCATATTGCTTGTGCTAG | AAAGTAGAACTCCAAAAGCATCTGA | 276 | 47467836 | 47468111 |
| HSTAL.232 | CCTGGAGTGGGTGAATCTTT | CTCATCTTCCAGGAACTGCC | 577 | 47467898 | 47468474 |
| HSSIL_GAP/M26B | CCATTCTCAGAGGCAATCACTGTAA | CCAAGAGGTAATGATCTCCAAGGTA | 189 | 47470259 | 47470447 |
| HSSIL_GAP/M26A | GAATTCACTTGCCTAACCACAGAA | TCAATTTATTACTATGTGCTTGTACTGCC | 438 | 47470505 | 47470942 |
| HSSIL/M47A | TACTAAGGCTTTTTAATGTATGGCTG | TCCAAACTCATTTGCAGTCATTTC | 551 | 47470810 | 47471360 |
| HSSIL_GAP/M25B | CTTTTAGTTGGCTGTCTGGAATTAC | ATTCCTTGGTATTTCCAGGTATACTG | 305 | 47470939 | 47471243 |
| HSTAL.236 | TGGCTTTTATCAGCCATACATT | GGGAACCCATGTAAGGGAAT | 508 | 47471325 | 47471832 |
| HSSIL_GAP/M25A | TAAAGTGACCAGTATGGAAAGTGAACA | ACAGGTAGTCAGAAAGAGAGAAGGCT | 295 | 47471671 | 47471965 |
| HSTAL.237 | TGTTCACTTTCCATACTGGTCA | CACCCTAAAGAACTCAGGGC | 593 | 47471939 | 47472531 |
| HSSIL_GAP/M23A | GTGCAAGACAACGTTCATTTGAAT | ACATTTGAACATTTGGCTGATAAGG | 257 | 47473407 | 47473663 |
| HSTAL.238 | TCCCACCAATGGTAGAGAATAA | TATGGCACATGGAAGGTTGA | 559 | 47473459 | 47474017 |
| HSTAL.239 | CCTTCCATGTGCCATATTCC | CATCAGTGGTACTGCCAAGG | 564 | 47474002 | 47474565 |
| HSSIL_GAP/M22A | AAATTTATCAGATGGGCAAGACATG | GTGATATAAAGCCTTGGCAGTACCA | 191 | 47474535 | 47474725 |
| HSTAL.240 | GCCTTGGCAGTACCACTGAT | GGAAATCATGGAAAGCCTGA | 532 | 47474545 | 47475076 |
| HSTAL.241 | TCAGGCTTTCCATGATTTCC | CCTATTCTCCTTGCCACTTCC | 446 | 47475057 | 47475502 |
| HSSIL_GAP/M21A | TTTGCCAAAGTACACTCAATTCCA | CGCCCAGCAGTCACAGATG | 332 | 47475546 | 47475877 |
| HSTAL.242 | AAAATCCCATGCTCCACTTG | GGGTGGAGTTTCCTCACAGA | 508 | 47475656 | 47476163 |
| HSSIL_GAP/M20A | AACCCAAGGGAGGCTAGAGAATT | TGGAGTCACTGGGAAAGGAAAC | 385 | 47475913 | 47476297 |
| HSTAL.243 | TTTTTGTAGGTGGTGGCACA | TCCTTGGACTGTGGTAAGCTG | 419 | 47477396 | 47477814 |
| HSSIL_GAP/M19A | ATGTAGCAAAGATTCCTTGGACTGT | TGTAGGTGGTGGCACAACTTAAA | 428 | 47477400 | 47477827 |
| HSSIL_GAP/M18B | TCCCCAATAAGACTGAAGTGCTG | GTGCCCATATCGCCAAAGTT | 228 | 47477887 | 47478114 |
| HSTAL.244 | GCCCATATCGCCAAAGTTTA | ATTCTCTGAGGCTCAATCGG | 598 | 47477889 | 47478486 |
| HSSIL/M31A | TTGTCTGCCAGCCTGATGAAT | TTCCGATTGAGCCTCAGAGAA | 322 | 47478465 | 47478786 |
| HSSIL_GAP/M18A | TGTCTGCCAGCCTGATGAAT | TTCCGATTGAGCCTCAGAGAATC | 321 | 47478465 | 47478785 |
| HSTAL.245 | CCGATTGAGCCTCAGAGAAT | TGAATTTGTGGGCAGTCAGA | 524 | 47478467 | 47478990 |
| HSSIL_GAP/M17B | GGGACTTTTTGATTCCATGCA | ACTGCCCACAAATTCAGCAA | 373 | 47478975 | 47479347 |
| HSSIL_GAP/M17A | ATTCGGCCAACTATTGTATTTGCT | AGTCCCCAGGAAGAGAAGCAGT | 403 | 47479342 | 47479744 |
| HSTAL.247 | GACCAGGATCAAAGCGATCT | TGGTTCTCTGACAGCAGACG | 553 | 47479396 | 47479948 |
| HSSIL_GAP/M15A | CAAGAAAGAAAGTATTCGGGTTCAA | TTAGATGGCAGCTAAGCACAGC | 210 | 47481287 | 47481496 |
| HSSIL_GAP/M14B | TTGTGGAACGTTGAGAAGCTTG | GCTAGGCTGACTCAAGGCTCTC | 219 | 47482073 | 47482291 |
| HSSIL/M27A | CAACGCCAACTGGAGATTTCAT | GCTCTCCCCGCCATAATCTAT | 538 | 47482089 | 47482626 |
| HSSIL_GAP/M14A | GTACCTTTCCACTTTCCTCCATCA | TCCACAAAGACACCATGCTGAC | 385 | 47482285 | 47482669 |
| HSTAL.250 | ACCAGGAGAATAGGCACTCAAA | TTTGCTTTTCATAACTGTTGGG | 548 | 47483767 | 47484314 |
| HSSIL_GAP/M12A | TGTGGTTTTCTCGTGTTATACACTCAT | TGAACTGATTTCCAGATAATTGTTAGATAATAC | 282 | 47484261 | 47484542 |
| HSSIL/M22B | TGGTTGGGTTCCTTCCCTCT | GGCAGGGGGGTAGAACACA | 412 | 47487145 | 47487556 |
| HSSIL_GAP/M9A | GGTTCCTTCCCTCTTACAAATACTGA | CAGGGGGGTAGAACACAAGG | 404 | 47487147 | 47487550 |
| HSTAL.252 | CCAACCAATTTCCCACTTGT | TTCACCAGACATCATGGAGC | 475 | 47487550 | 47488024 |
| HSSIL_GAP/M8A | GCCCCAAATAAATATCTAGCCCA | GGGCCGTGCAAAAGGATATA | 475 | 47487982 | 47488456 |
| HSTAL.255 | ATCCTCCACTCCCATTGCTT | AGATGGAACTGTCCAGTGGG | 445 | 47488878 | 47489322 |
| HSTAL.256 | CCCACTGGACAGTTCCATCT | GCACGTTTTTCTCTGTCTTGAA | 580 | 47489303 | 47489882 |
| HSTAL.257 | TTCAAGACAGAGAAAAACGTGC | CCATCTTTTCACCCAAATGAA | 541 | 47489861 | 47490401 |
| HSSIL/M19A | TAAGGTGGTGGGTATGGAAAGATAA | AGCTTCCACAGAATGCAGGC | 409 | 47490209 | 47490617 |
| HSSIL_GAP/M6A | TCTTCTAGCAGCATTATGGGGACT | CCACAGAATGCAGGCTTTAAATC | 464 | 47490214 | 47490677 |
| HSTAL.258 | CATACCCACCACCTTAGTTGC | GCATCCTGTGCAGATATTGAAA | 600 | 47490602 | 47491201 |
| HSSIL_GAP/M5B | ACGGTGGGAATTTCTTGAGGACT | ATGCCTCCCTTAACTTGGAATG | 291 | 47491198 | 47491488 |
| HSSIL_GAP/M5A | CGCCCGCAGTTCTCCAAG | AGCTCAGATGATACCCAAGGATTTC | 497 | 47491542 | 47492038 |
| HSSIL/M18A | CGGGTGTCCGCTTCCAGT | CGCGGAGCTGAGGTCTGTT | 495 | 47491737 | 47492231 |
| HSSIL_GAP/M4A | TGTCACTAAAACAGATGGCTTCCTAG | AGGATCCGAGGATTTAAACCTTT | 360 | 47492059 | 47492418 |
| HSTAL.261 | TTCCTCAACACTCCACTTTTGT | CAGGAAAGCAGAGAATGATGC | 509 | 47495016 | 47495524 |
| HSTAL.262 | GCATCATTCTCTGCTTTCCTG | CATGTAACCAATGGCCGTC | 401 | 47495504 | 47495904 |
| HSTAL.263 | CAAGGTGAGAGCTGAGGCAT | GGGTCAGATCCCCAAAGCTA | 600 | 47499089 | 47499688 |
| HSSIL/M10A | TCAGTTCCCTTAATCTCTTTGAACA | ACCACTGGACTTCTAAGCAGGATAT | 417 | 47499474 | 47499890 |
| HSTAL.264 | GTGCTAGCTGGTATGGGGAG | GCTTTTCTCTGTAAGGGCCA | 573 | 47500449 | 47501021 |
| HSTAL.265 | TGGCCCTTACAGAGAAAAGC | TCATGTGGACAGGGACTGAA | 584 | 47501002 | 47501585 |
| HSTAL.266 | TCACCAGGAACAGGGAAGTT | GGAATTTGGCATAAATTGACAG | 558 | 47501683 | 47502240 |
| HSSIL/M7A | ACGCCCAGCCCATCTCAA | CAATTTATGCCAAATTCCTTTTAGAC | 431 | 47502223 | 47502653 |
| HSSIL/M5A | CCCACAAGGCAATAGATGACAA | TAGGCAGCAAAGGGGAGTAGTC | 418 | 47504210 | 47504627 |
| HSTAL.269 | TCATCTATTGCCTTGTGGGG | TACAGCCTTTCCAGTTGGGT | 449 | 47504609 | 47505057 |
| HSTAL.270 | ACCCAACTGGAAAGGCTGTA | TGCTAGATTGTGGGGAGGTC | 419 | 47505038 | 47505456 |
| HSTAL.271 | GACCTCCCCACAATCTAGCA | TGTCTACTATGTGCCAGGCATT | 426 | 47505437 | 47505862 |
| HSTAL.272 | ATGCCTGGCACATAGTAGACA | TTAGTGCTCCTTGGAGGTGG | 505 | 47505842 | 47506346 |
| HSSIL/M3A | CAAAAGCCCTCTGTGGAACCT | GAAATGCCCCTTTACTGGGTG | 512 | 47506217 | 47506728 |
| HSTAL.273 | TGATCAAATAGGCCCAAAGC | TAGAGACGGGAAGCTGGAAA | 566 | 47506516 | 47507081 |
| HSSIL/M2A | ACATGGGCAGCCAAGAACAA | ATGGGTGTCTGGGTTTCAGTTTT | 475 | 47508028 | 47508502 |
| HSSIL/M33A | TTTGGGGACCAGATTCGTCTC | AGGCTGGGAGACAGGAAGTGA | 471 | 47512062 | 47512532 |
| HSTAL.277 | CACTGTCACCGAACGTGTCT | TGGTCGCCTCTTCTGTTTCT | 515 | 47512283 | 47512797 |
| HSSIL/M34A | GGTTGGTTAGCTGCATTGACACT | AAATACATCTGTCCAACACATAATGACT | 342 | 47512690 | 47513031 |
| HSTAL.278 | GCACACAGAGACAGATGCAGA | TGCATTCAAATATAACACGCAT | 484 | 47514101 | 47514584 |
| HSTAL.279 | AATTGCTATTCCCTAGCTGGC | AATTTCAGGTGCCCTGTGAG | 562 | 47515265 | 47515826 |
| HSSIL/M37A | CATAAATCCACGGTAAATTCTCCA | AATCCCAAAACCTCCCTCCA | 382 | 47515758 | 47516139 |
| HSTAL.281 | GGTTTTGGGATTGATTTTGC | GAAAGCAATTTGGCATGGTT | 595 | 47516128 | 47516722 |
| HSTAL.282 | TGCTTTCTGTAGCACCCAGA | CCTCTGGGGATCGAATTTTT | 591 | 47516716 | 47517306 |
| HSTAL.283 | TGAGCAGATTTCCAAGCTCC | CTGAAGATCTCCCCTCCTCC | 559 | 47518205 | 47518763 |
| HSSIL/M38A | ATTAAGGGTGCTTATGACATGGG | TGAGAAAGAGATGGGAAAGCAGTT | 220 | 47518704 | 47518923 |

Supplementary Table S.2
