# Supplementary material for: Genomic Approaches Uncover Increasing Complexities in the Regulatory Landscape at the Human SCL (TAL1) Locus
Source: PLoS One. 2010 Feb 5;5(2):e9059. doi: 10.1371/journal.pone.0009059 (PMC2816701; doi:10.1371/journal.pone.0009059)
Supplement: Table S3 — Antibodies used for ChIP-chip assays performed in this study. Antibodies for histone modifications, transcription factors, histones and antisera controls are listed along with their supplier and catalogue numbers. (0.05 MB DOC) [file pone.0009059.s012.doc]

|  | **Antibody** | **Catalogue Number** | **Supplier** |
| --- | --- | --- | --- |
| **Histone Modifications** |  |  |  |
|  | histone H3 K4me1 | ab8895 | Abcam |
|  | histone H3 K4me2 | ab7766 | Abcam |
|  | histone H3 K4me3 | ab8580 | Abcam |
|  | histone H3 K27me3 | 07-449 | Millipore (Upstate) |
|  | histone H3 K9/K14ac | 06-599 | Millipore (Upstate) |
|  |  |  |  |
| **Transcription Factors** |  |  |  |
|  | GATA1 | sc-1234X | Santa Cruz Biotechnology |
|  | SCL(TAL1) |  | a gift from D. Mathieu-Mahul (Montpellier, France) |
|  | LDB1 | sc-11198X | Santa Cruz Biotechnology |
|  | E2A (E47) | sc-763 | Santa Cruz Biotechnology |
|  | E2A (E12) | sc-762 | Santa Cruz Biotechnology |
|  | LMO2 | sc-10497 | Santa Cruz Biotechnology |
|  | CTCF | sc-15914 | Santa Cruz Biotechnology |
|  | RNA pol II | ab817 | Abcam |
|  | TAFII 250 | sc-17134 | Santa Cruz Biotechnology |
|  |  |  |  |
| **Histones** |  |  |  |
|  | histone H3 | ab1791 | Abcam |
|  | histone H2B | ab1790 | Abcam |
|  |  |  |  |
| **Antisera Controls** |  |  |  |
|  | rabbit IgG | 12-370 | Millipore (Upstate) |
|  | goat IgG | sc-2028 | Santa Cruz Biotechnology |
|  | mouse IgG | 12-371 | Millipore (Upstate) |

Supplementary Table S.3
