# Supplementary material for: Genomic Approaches Uncover Increasing Complexities in the Regulatory Landscape at the Human SCL (TAL1) Locus
Source: PLoS One. 2010 Feb 5;5(2):e9059. doi: 10.1371/journal.pone.0009059 (PMC2816701; doi:10.1371/journal.pone.0009059)
Supplement: Table S4 — Oligonucleotide primer pairs used to PCR amplify the regions cloned into the pGL3 luciferase reporter constructs. Construct names in the first column are as described in Figure 4. The BamHI, Kpn1 and HindIII sites added to the sequence of each primer are shown in brackets. Amplicon sizes and genomic sequence co-ordinates are from NCBI build 35. (0.03 MB DOC) [file pone.0009059.s013.doc]

| **Amplicon Name** | **Primer 1 (5'→3')** | **Primer 2 (5'→3')** | **Amplicon Size (bp)** | **Chrom 1 Co-ordinate Start** | **Chrom 1 Co-ordinate Finish** |
| --- | --- | --- | --- | --- | --- |
| SCL pro1a | (GGAAGCTTC)GCCAGAATGCTCACGTTTTT | (GGGGTACCC)GGGAGGTGACGATTTTGGTA | 548 | 47409845 | 47410392 |
| -31 | (CGCGGATCC)TCTCAAGGGCACAGAGTGTCA | (CGCGGATCC)ATCCTCTTGCTCAGCCTCCTG | 1008 | 47440142 | 47441149 |
| -13 | (CGGGATCCCG)CAGGAGCATTCAGCAGATCT | (CGGGATCCCG)TGACGTTTGCAGACTCCTTCA | 2235 | 47420799 | 47423033 |
| -10 | (CGCGGATCC)GAACACCACGTGAACCTCCTC | (CGCGGATCC)AAGGCCACACAGGTGAATTA | 900 | 47418981 | 47419880 |
| -7 | (CGGGATCCCG)TTGACCTCAGATGATCCGCC | (CGGGATCCCG)TCATTTCCCCTTCTCCCCAG | 952 | 47416433 | 47417384 |
| +51 | (CGGGATCCCG)AAAGGTTGGGAAGAGAGCAGG | (CGGGATCCCG)GGGTCAGGCCTCTGCTAAGG | 936 | 47358744 | 47359679 |
| +57 | (CGCGGATCC)ACAGCCAGCCAGGATGCTT | (CGCGGATCC)TGACTCCACCTCCTTCTCCC | 724 | 47352493 | 47353216 |
| enhancer assay  negative control | (CGGGATCCCG)ACTTTCCTTTTAAGTACACCAGCAAC | (CGGGATCCCG)TTTCAATGAGCTATTGGATTATGTG | 951 | 47289370 | 47290320 |
| SCL+53d | (CGCAAGCTT)CAATTTTACCGGCAAAGGAA | (CGCGGTACC)GCATCCCTTTTCAGTTTCCA | 971 | 47357585 | 47358555 |
| SCL+53u | (CGCGGTACC)AATTTTACCGGCAAAGGAA | (CGCAAGCTT)CGCATCCCTTTTCAGTTTCCA | 971 | 47357585 | 47358555 |

Supplementary Table S.4
