# Supplementary material for: Genomic Approaches Uncover Increasing Complexities in the Regulatory Landscape at the Human SCL (TAL1) Locus
Source: PLoS One. 2010 Feb 5;5(2):e9059. doi: 10.1371/journal.pone.0009059 (PMC2816701; doi:10.1371/journal.pone.0009059)
Supplement: Table S5 — Oligonucleotide primer pairs used to PCR amplify the regions cloned into the enhancer blocking reporter constructs. Construct names in the first column are as described in Figure 4. The AscI sites added to the sequence of each primer are shown in brackets. Amplicon sizes and genomic sequence co-ordinates are from NCBI build 35. (0.03 MB DOC) [file pone.0009059.s014.doc]

| **Amplicon Name** | **Primer 1 (5'→3')** | **Primer 2 (5'→3')** | **Amplicon Size (bp)** | **Chrom 1 Co-ordinate Start** | **Chrom 1 Co-ordinate Finish** |
| --- | --- | --- | --- | --- | --- |
| -31 | (TAGGCGCGCCTA)CTCAAGGGCACAGAGTGTCA | (TAGGCGCGCCTA)ACTAGCTGAGCATGGTGGTG | 1470 | 47440143 | 47441612 |
| +40 | (TAGGCGCGCCTA)AGGAGGAAACAGGAGGAAGC | (TAGGCGCGCCTA)GCTCTCTGGCTTACAGCAATG | 1462 | 47370030 | 47371491 |
| +53 | (TAGGCGCGCCTA)CAGAAAAGCTCCCGAAACTG | (TAGGCGCGCCTA)CCTGCCTCCTAAGCTTCCTT | 1466 | 47357035 | 47358500 |
| +57 | (TAGGCGCGCCTA)TGGATTCAACCCTTAAGGAAAA | (TAGGCGCGCCTA)CCCTCAGTCCCTGAGTTTCA | 1493 | 47352207 | 47353699 |

Supplementary Table S.5
