# Supplementary material for: Genomic Approaches Uncover Increasing Complexities in the Regulatory Landscape at the Human SCL (TAL1) Locus
Source: PLoS One. 2010 Feb 5;5(2):e9059. doi: 10.1371/journal.pone.0009059 (PMC2816701; doi:10.1371/journal.pone.0009059)
Supplement: Table S6 — Oligonucleotide primer pairs used to perform SyBr green quantitative real-time PCR for regions enriched for histone H3 K9/K14ac. Amplicon names in the first column are as shown in Figure S2 (panel a) while the second column lists alternative names which describe corresponding amplicons found in Table S2. Amplicon sizes and genomic sequence co-ordinates are taken from NCBI build 35. (0.12 MB DOC) [file pone.0009059.s015.doc]

| **Region Name** | **Amplicon Name** | **Primer 1 (5'→3')** | **Primer 2 (5'→3')** | **Amplicon Size (bp)** | **Chrom 1 Co-ordinate Start** | **Chrom 1 Co-ordinate Finish** |
| --- | --- | --- | --- | --- | --- | --- |
| KCY +1 | HSTAL.277q | TGGTTGGTTAGCTGCATTGAC | CCTTCTTTCCCAAGCATTCC | 75 | 47512689 | 47512763 |
|  | HSSIL/M33Aq | GCGCAGAGGTTAGCGTGTC | GCCTCTAACCCAAATCCGC | 71 | 47512136 | 47512206 |
| KCY -4 | HSSIL/M2Aq | AGTCTTTGTTGTTTCCATGATAGAAC | GGTTTACTAGGAAGTTTCAGACCC | 80 | 47508213 | 47508292 |
| NC i | HSSIL/M10Aq | TCTCTTTGAACACAGGGCAATG | TATTAGTCTAGGTGTACTGGCAGTTG | 71 | 47499807 | 47499877 |
| SIL -1/+1 | HSSIL/GAP/M4Aq | CAGTCGCCGACCAATGATC | GCTAGGTAGACGGAGGAGCG | 73 | 47492145 | 47492217 |
|  | HSSIL_GAP/M5Aq | GCTCCTACCCTGCAAACAGAC | GGAAACCAGGAGCACAAAGC | 71 | 47491699 | 47491769 |
| NC ii | HSSIL/M51Bq | TGAATGCTTCCCTTGTGATG | GTAATGTTTCCTTACTGGTTAGCAAC | 71 | 47467138 | 47467208 |
| NC iii | HSSCL/M15Bq | GTGCCCTTGAGAGCCTAGGG | CCTCAACAGCCTGTCTTATAATTG | 71 | 47440083 | 47440153 |
| SCL -9/-10 | HSTAL.175q | GGCCAGAGTTCAAATCCTGAC | CAAGCGTAAAGTGACATGCCC | 71 | 47419831 | 47419901 |
|  | HSSCL/M36Bq | AGAGGAAGGACCTTCAGCTCC | TCCTCAAGGCAGAGAGAGCC | 71 | 47418997 | 47419067 |
| SCL +1 | HSSCL/M46Aq | TTCCCCCTTTTCCTTACGC | CGCACTCTCACAATCCCACC | 102 | 47409535 | 47409636 |
|  | HSSCL/M46Bq | CCGTTGGTGTTCTCAGCAGG | CACCCAAACACAGTCGCAG | 71 | 47409309 | 47409379 |
| SCL +2 | HSSCL/M47Aq | AGGCCCCGAAAGGACAATG | CAGGAGGTGATCCCGAATG | 73 | 47408628 | 47408700 |
|  | HSSCL/M47Bq | AGCCGTTTTCTAAGTTGCTGG | CCAAGTCTCTGTGTCCGTGC | 71 | 47408265 | 47408335 |
| SCL +3 | HSTAL.157q | TTTCGAACCCTCCAACTGG | CAACCGCGTAGACACCTCC | 72 | 47407338 | 47407409 |
|  | HSSCL/M48Aq | ACTGAACCAGACCGATCCCAG | AACAACAACCCCTCCCGAC | 71 | 47407303 | 47407373 |
| SCL +4/+5 | HSSCL/M48Bq | ACCGCAGCGTAACTGCAGG | CGAGGAAGAGGATGCACACC | 73 | 47406956 | 47407028 |
|  | HSTAL.156q | GAAGCCGAGGAAGAGGATGC | CGTAACTGCAGGCCTCTCAG | 71 | 47406951 | 47407021 |
|  | HSSCL/M49Bq | GGAGAGGACATTTGTGGCCAG | CGGTGGATTCCTGAGAGGC | 71 | 47405741 | 47405811 |
| SCL +8/+9 | HSSCL/M53Aq | CAGTCAATGAACCTGGCGG | CCTAGCTCTCTGCCCTCACC | 73 | 47402359 | 47402431 |
|  | HSSCL/M53Bq | CACCCTGCAGCTGAGCTAGG | TGCAGGACTGTGAGTGTGGTC | 71 | 47401484 | 47401554 |
| NC iv | HSTAL.138q | CATCACCTGCAAAATGGAGG | TAAGCTGAGGCAGGCATTGTC | 103 | 47398315 | 47398417 |
| SCL +19 | HSSCL/M63Aq | CACGTGCGATCTATCTCTTCG | GGCAAATGCTGAAAGGAACC | 71 | 47391506 | 47391576 |
|  | HSSCL/M64Aq | TCTGCTGTAGCCATGGTCCTG | GCAACAAAAGCAGTGCAAGG | 71 | 47391323 | 47391393 |
| SCL +20/+22 | HSTAL.122q | CCCAGTGGTCTGACTCCAAAG | GCACAGAAGGCAGTGAATGG | 74 | 47390543 | 47390616 |
|  | HSSCL/M65Aq | GATACAGAGCCCTTCCACCC | CAGAGCAGGATCTCCCGTG | 74 | 47390110 | 47390183 |
|  | HSTAL.121q | TTCGAACGGATCACATCCTG | TTGGTCCGAGCTCTGCCTC | 76 | 47389763 | 47389838 |
|  | HSSCL/M66Aq | CCTCACCTCTAGGCAGCCAG | GCTTTGGATCAGACACACGTG | 71 | 47389401 | 47389471 |
|  | HSTAL.119q | CTTTGCAGCATTCAAGGCC | GCAGCTGGTAAGGCACCTG | 71 | 47388329 | 47388399 |
|  | HSSCL/M67Aq | CGAGGCTGCTTAGAGAGAGGC | GGACCATAGCACCGCAGTC | 101 | 47388075 | 47388175 |
| NC v | HSTAL.108q | GGATTGAGGAGAGGGCATGTG | GCACGGCTGTGGAGCTATG | 101 | 47377642 | 47377742 |
| NC vi | HSTAL.106q | CAGCAGAGGTCCCAAAGCC | CAGTACTCCCAGCTTGCTTCC | 101 | 47374561 | 47374661 |
| SCL +43/+45 | HSSCL/M87Aq | CTTTCCCTAGAATCCAGCCCC | GGAGAGTCCCAGCCTCACC | 71 | 47367938 | 47368008 |
|  | HSTAL.98q | GCATTCGAGGTCATCTTCCAG | GGACTCTCCCACATCTGCTTG | 71 | 47367876 | 47367946 |
|  | HSSCL/M88Aq | GCCTGTCACTTGTTTTCAACG | CAAATCCTGTTCCTCCCTGAG | 73 | 47367324 | 47367396 |
|  | HSSCL/M88Bq | TGTCTCCCAGGTCTTGGAAGC | TCTTGCCGTGCTCTGTGAC | 71 | 47366737 | 47366807 |
|  | HSTAL.95q | TGGCTCACTCCTGCTCAAATG | GAGAAGAGGTCAGGCCTCCAC | 78 | 47366287 | 47366364 |
|  | HSSCL/M90Aq | GGTCCCCAAGACCCAGAGTAG | CAGAGAGAGGAGTGGACCAGG | 76 | 47365683 | 47365758 |
|  | HSTAL.93q | GCCCATCTGACCCACTTATGC | TGTTGTTCCTCCAGCTCCC | 71 | 47365279 | 47365349 |
| SCL +51 | HSTAL.84q | TTAAGCCGAAGCCCAGAGAG | GCTCCAGGCCTATCCTTGC | 71 | 47359910 | 47359980 |
|  | HSSCL/M96Aq | CACATTTCCTCCCAGCTCTGC | GGCTGGTGGAGTGACCTGAC | 76 | 47359505 | 47359580 |
|  | HSSCL/96Bq | TGACCTTACAGCCCTTCACCC | AGCTCCCTGCTCCCAGCAC | 72 | 47359190 | 47359261 |
| SCL +52/+53 | HSTAL.82q | TGGAGGAGGAGAAAGGCAAAC | CCATCCATCTCTGTCTCCCTG | 96 | 47358590 | 47358685 |
|  | HSSCL/M97Aq | GCTCTCAGCCCAGAATGTCC | CAAGGTGCAAGCCCTGTTC | 71 | 47358287 | 47358357 |
|  | HSSCL/M98Aq | TCCATGATCAGCGTAGATGCC | GGAGGAAGTGCTGAACCCAG | 71 | 47357425 | 47357495 |
| NC vii | HSTAL.77q | TTCTGTACCTGCCAGCCAAG | CCCGACGAGCGTTATGTAAG | 71 | 47355954 | 47356024 |
| NC viii | HSSIL/M55Aq | TCATGATGATATTTAGCATACTCAGCAAAG | GGAGAATGATAACTTGTGTCAGGC | 91 | 47342423 | 47342513 |
| NC ix | HSSCL/M137Aq | TCTCTGGAAGTCATAAATACAACA | AATCTGCTCATCAAGTAATACG | 71 | 47318153 | 47318223 |
| NC x | HSSCL/M182Aq | TTTGCAGTGCCCTGTTCTTAG | TGTTGGCTACCTTGATCATGTG | 71 | 47273688 | 47273758 |
| NC xi | HSTAL.7q | TCATGCCATTTCCGTTGTAC | TTGAACACTTGGAGATGATGATG | 71 | 47273478 | 47273548 |

Supplementary Table S.6
