# Supplementary material for: Genomic Approaches Uncover Increasing Complexities in the Regulatory Landscape at the Human SCL (TAL1) Locus
Source: PLoS One. 2010 Feb 5;5(2):e9059. doi: 10.1371/journal.pone.0009059 (PMC2816701; doi:10.1371/journal.pone.0009059)
Supplement: Table S7 — Oligonucleotide primer pairs used to perform SyBr green quantitative real-time PCR for regions enriched for GATA1. Amplicon names in the first column are as described in Figure S2 (panel b) while the second column lists alternative names which describe corresponding amplicons found in Table S2. Amplicon sizes and genomic sequence co-ordinates are from NCBI build 35. (0.04 MB DOC) [file pone.0009059.s016.doc]

| **Amplicon Name** | **Alternative Name** | **Primer 1 (5'→3')** | **Primer 2 (5'→3')** | **Amplicon Size (bp)** | **Chrom 1 Co-ordinate Start** | **Chrom 1 Co-ordinate Finish** |
| --- | --- | --- | --- | --- | --- | --- |
| NC i | HSTAL.108q | GGATTGAGGAGAGGGCATGTG | GCACGGCTGTGGAGCTATG | 101 | 47377642 | 47377742 |
| NC ii | HSSCL/M182Aq | TTTGCAGTGCCCTGTTCTTAG | TGTTGGCTACCTTGATCATGTG | 71 | 47273688 | 47273758 |
| NC iii | HSSCL/M15Bq | GTGCCCTTGAGAGCCTAGGG | CCTCAACAGCCTGTCTTATAATTG | 71 | 47440083 | 47440153 |
| NC iv | HSSIL/M5Aq | CAGGGTATATCTATGTTCCTAGCAC | GATTGATGAATGGTGACAAAGC | 71 | 47504296 | 47504366 |
| SCL -9/-10a | HSSCL/M36Aq | GGCCTGTGCTGTGACTCTCC | CCACAGATGGTCTCTGTGCTG | 71 | 47419540 | 47419610 |
| SCL +7/+8 | HSSCL/M53Aq | CAGTCAATGAACCTGGCGG | CCTAGCTCTCTGCCCTCACC | 73 | 47402359 | 47402431 |
| SCL -9/-10b | HSTAL.175q | GGCCAGAGTTCAAATCCTGAC | CAAGCGTAAAGTGACATGCCC | 71 | 47419831 | 47419901 |
| SCL -7 | HSSCL/M38Bq | TTTGTGCCCTGTGTGCCTG | TGATTAGCATACCCTGGAGCC | 73 | 47416835 | 47416907 |
| SCL Prom 1a | HSSCL/M45Aq | GGATAGGGAGACTGCCCATTG | CACCTCCCAGGGCTTCTTTC | 76 | 47410385 | 47410460 |
| SCL +51 | HSSCL/M96Bq | TGACCTTACAGCCCTTCACCC | AGCTCCCTGCTCCCAGCAC | 72 | 47359190 | 47359261 |

Supplementary Table S.7
