# Supplementary material for: Genomic Approaches Uncover Increasing Complexities in the Regulatory Landscape at the Human SCL (TAL1) Locus
Source: PLoS One. 2010 Feb 5;5(2):e9059. doi: 10.1371/journal.pone.0009059 (PMC2816701; doi:10.1371/journal.pone.0009059)
Supplement: Table S8 — Oligonucleotide primer pairs used to perform SyBr green quantitative real-time PCR to detect reverse-transcribed cDNA for transcripts a, b and c of unknown function. Amplicon for transcripts a and b was from coding sequence shared by both transcripts - thus one amplicon represents both transcripts. The primer pairs for SCL and β-actin are also shown. Alternative names for the primer pairs are shown. Amplicon sizes and genomic sequence co-ordinates are taken from NCBI build 35. n.a. = not applicable. (0.03 MB DOC) [file pone.0009059.s017.doc]

| **Amplicon Name** | **Alternative Name** | **Primer 1 (5'→3')** | **Primer 2 (5'→3')** | **Amplicon Size (bp)** | **Chrom 1 Co-ordinate Start** | **Chrom 1 Co-ordinate Finish** |
| --- | --- | --- | --- | --- | --- | --- |
| SCL | TAL1 | TTTTGTGAAGACGGCACGG | TGAGAGCTGACAACCCCAGG | 135 | 47397053 | 47397187 |
| Transcript a/b | RP1-18D14.4-001 (a) ENSESTT00000094074 (b) | GACAGAAAAGCTCCCGAAAC | ATGCGCATATGCTCTGTCTC | 96 | 47357033 | 47357128 |
| Transcript c | RP1-18D14.3-001 | CGACGAGCGTTATGTAAGGA | TGATGCCTCAAGAGATCCAG | 113 | 47355910 | 47356022 |
| β-actin | n.a. | AGAAGGAGATCACTGCCCTGG | CACATCTGCTGGAAGGTGGAC | 127 | n.a. | n.a. |

Supplementary Table S.8
